# Cytoplasmic anillin and Ect2 promote RhoA/myosin II-dependent confined migration and invasion

**DOI:** 10.1038/s41563-025-02269-9

**Published:** 2025-06-26

**Authors:** Avery T. Tran, Emily O. Wisniewski, Panagiotis Mistriotis, Konstantin Stoletov, Maria Parlani, Alice Amitrano, Brent Ifemembi, Se Jong Lee, Kaustav Bera, Yuqi Zhang, Soontorn Tuntithavornwat, Alexandros Afthinos, Alexander Kiepas, Bhawana Agarwal, Sanjiban Nath, John J. Jamieson, Yi Zuo, Daniel Habib, Pei-Hsun Wu, Stuart S. Martin, Sharon Gerecht, Luo Gu, John D. Lewis, Petr Kalab, Peter Friedl, Konstantinos Konstantopoulos

**Affiliations:** 1 Department of Chemical and Biomolecular Engineering, The Johns Hopkins University, Baltimore, MD USA; 2 Johns Hopkins Institute for NanoBioTechnology, The Johns Hopkins University, Baltimore, MD USA; 3 Department of Chemical Engineering, Auburn University, Auburn, AL USA; 4 Department of Oncology, University of Alberta, Edmonton, AB Canada; 5 Department of Medical Biosciences, Radboud University Medical Center, Nijmegen, Netherlands; 6 Department of Materials Science and Engineering, The Johns Hopkins University, Baltimore, MD USA; 7 Marlene and Stewart Greenebaum National Cancer Institute Comprehensive Cancer Center, University of Maryland School of Medicine, Baltimore, MD USA; 8 Department of Pharmacology, University of Maryland School of Medicine, Baltimore, MD USA; 9 Department of Biomedical Engineering, Duke University, Durham, NC USA; 10 Department of Genitourinary Medicine, UT MD Anderson Cancer Center, Houston, TX USA; 11 Department of Biomedical Engineering, The Johns Hopkins University, Baltimore, MD USA; 12 Department of Oncology, The Johns Hopkins University, Baltimore, MD USA; 13 Present Address: Department of Chemical Engineering, Mahidol University, Nakhon Pathom, Thailand

**Keywords:** Cell invasion, Cancer models

## Abstract

Cell migration in mechanically confined environments is a crucial step of metastatic cancer progression. Nonetheless, the molecular components and processes mediating such behaviour are still not fully understood. Here we demonstrate that a pool of the scaffolding protein anillin and its cofactor Ect2, which are both predominantly nuclear proteins and critical mediators of cytokinesis, is present in the cytoplasm of multiple interphase cell types that promote confined cell migration. Confined migration in biomimetic microfluidic models triggers the actomyosin-binding-dependent recruitment of anillin to the plasma membrane at the poles of migrating cells in a manner that scales with microenvironmental stiffness and confinement. The guanine nucleotide exchange activity of Ect2 is required for its RhoA-GTPase-mediated activation of myosin II at the cell poles, enhancing invasion, bleb-based migration and extravasation. Confinement-induced nuclear envelope rupture further amplifies this process due to the release of further anillin and Ect2 into the cytoplasm. Overall, these results show how Ect2 and anillin cooperate to mediate RhoA/ROCK/myosin II-dependent mechanoadaptation and invasive cancer progression.

## Abstract (web summary)

Cell migration in confined environments is initiated by a cytoplasmic pool of anillin and Ect2 that promotes RhoA/myosin II-mediated activation at the poles of migrating cells, in a process dependent on the extracellular environment stiffness.

## Main

Cancer cell migration through confined spaces is a critical step in the metastatic process^1^. To colonize distant organs, cells escaping from the primary tumour must successfully navigate pores in the extracellular matrix (ECM) as well as three-dimensional (3D) longitudinal tissue tracks, which exist naturally between various anatomical structures or can be created de novo by the matrix remodelling of dense ECM by stromal cells or the tumour cells themselves^1^. Such paths impose varying degrees of confinement, as cells must travel through confining pores ranging from 1 µm to 20 µm in diameter, or fibre- and channel-like tracks varying from less than 3 µm up to 30 µm in width^2^. During extravasation, tumour cells must also transmigrate through narrow gaps between adjacent endothelial cells ranging from 2 µm to 5 µm in diameter^3^. Physical cues, such as confinement, initiate intracellular signalling cascades that enable cells to adapt their migration mechanisms and modes to their microenvironment^4^.

Rho GTPases, which are overexpressed in human tumours^5^, play a pivotal role in regulating confined cell migration^1^. Although Rac1 promotes actin polymerization and the formation of lamellipodia protrusions typically associated with a mesenchymal migration mode that has commonly been observed on two-dimensional (2D) substrates, Rho GTPase signalling also regulates other distinct migration modes in 3D microenvironments^6^. Bleb-based migration, which is characterized by spherical membrane protrusions produced by contractions of the actomyosin cortex^7^, is one such migration phenotype prompted by RhoA/ROCK activity^8^ when the cell–matrix adhesion is low. Bleb-based migration is often induced by extracellular protease inhibition^8^, allowing cells to squeeze through gaps in the ECM^7^. Different amoeboid modes of migration are characterized by stable bleb formation^9^. In addition to bleb-based migration, elevated RhoA-dependent contractility promotes lobopodial-based migration by facilitating nuclear pulling^10^. Furthermore, the distinct spatial regulation of Rho GTPases can tune the migration mode in confinement^11^. In conjunction with Rac1-driven lamellipodia or filopodia formation at the cell leading edge, RhoA/ROCK orchestrates the translocation of the cell rear in matrix-directed cell migration^12^.

Besides confined cell migration, Rho GTPase regulation is essential during cell division, specifically during cytokinesis, when RhoA acts as a critical regulator of actomyosin-based contractile ring formation and ingression^13^. During cytokinesis, RhoA is directly activated by RhoGEF Ect2 (ref. ^14^), which gets anchored to the equatorial cortex via its interaction with anillin^15^, a scaffolding protein that binds to the plasma membrane and links RhoA, actin and myosin^16^. Several phenotypic parallels exist between RhoA/myosin II-dependent contractility activation during cytokinesis and confined cell migration, including polarized distributions of myosin II and actin; colocalization of RhoA, actin and myosin II (refs. ^11,16,17^); and cytoplasmic bleb formation^7,11^. In addition to their primary role in cell cytokinesis, recent evidence has implicated anillin and Ect2 in tumourigenic and metastatic processes^18,19^. Specifically, anillin is upregulated in different cancer types such as pancreatic, breast and lung cancers^18^, and its overexpression promotes anchorage-independent proliferation, wound healing and invasion into Matrigel^20^. Ect2 has been reported to be recruited by caveolae at the rear of fast-migrating fibroblasts and activate RhoA-mediated rear retraction^12^. Here we demonstrate that confinement promotes the recruitment of cytoplasmic anillin to the subcortical actomyosin domains at the front and rear poles of migrating cells, resulting in the formation of anillin-rich zones at cell edges, referred to as ACEs. Nuclear envelope (NE) rupture, which frequently occurs during cell entry and migration in confining spaces, further enriches anillin at the cell poles as well as cytoplasmic Ect2 that activates RhoA. Anillin functions as a critical scaffolding factor for recruiting active RhoA to the cell poles and locally activating actomyosin contractility. Taken together, anillin and Ect2 cooperate to promote RhoA/ROCK/myosin II contractility in migrating cells in confinement, thereby facilitating their invasion and extravasation.

## Confinement-induced RhoA activity promotes bleb-based motility

**Fig. 1:**
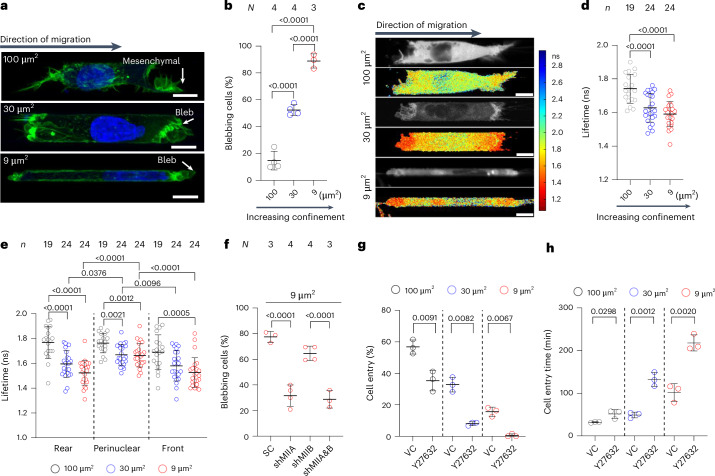
Confinement induces RhoA activation to facilitate cell entry and bleb-based migration. **a**, Representative confocal sections of HT-1080 cell migration phenotype in 100-µm^2^, 30-µm^2^ and 9-µm^2^ channels, as assessed from cells fixed and stained with AF488 phalloidin and Hoechst 33342. Scale bars, 10 µm. **b**, Percentage of HT-1080 cells migrating with a bleb-based migration phenotype in 100-µm^2^, 30-µm^2^ and 9-µm^2^ channels (*n* ≥ 20 cells per experiment from *N* experiments). **c**, Increasing confinement elevates RhoA activity and polarization as measured by FLIM–FRET of a RhoA2G biosensor. Representative cells in channels of the prescribed cross-sectional area. Greyscale images of donor intensity and pseudocolour images showing the subcellular distribution of activated RhoA were prepared with SymPhoTime 64 from single confocal sections acquired with the PicoQuant FLIM system. Scale bars, 10 µm. **d**, Donor fluorescence lifetime of RhoA activity biosensor RhoA2G inside 100-µm^2^, 30-µm^2^ and 9-µm^2^ channels, as measured by FLIM–FRET (*n* cells from *N* = 3 experiments). **e**, Spatial distribution of RhoA activity in cells migrating inside 100-µm^2^, 30-µm^2^ and 9-µm^2^ channels as measured by FLIM–FRET (*n* cells from *N* = 3 experiments). **f**, Percentage of scramble control (SC), MIIA, MIIB, or dual MIIA and MIIB knockdown HT-1080 cells migrating with a blebbing phenotype in 9-µm^2^ channels (*n* ≥ 15 cells per experiment from *N* experiments). **g**,**h**, Percentage of cells that enter (**g**) and time required for cell entry (**h**) into 100-µm^2^, 30-µm^2^ and 9-µm^2^ channels and in the presence of Y27632 (10 μM) or vehicle control (VC). Data are represented as averages per experiment (*n* ≥ 20 cells per experiment from *N* = 3 experiments). Values represent the mean ± s.d. Statistical significance was assessed by one-way ANOVA followed by Tukey’s multiple comparisons test (**b**, **d**, **e** (rear) and **f**), Kruskal–Wallis followed by Dunn’s multiple comparisons test (**e** (perinuclear and front)) and two-tailed unpaired *t*-test (**g** (100 µm^2^) and **h**) with Welch’s correction (**g** (30 µm^2^ and 9 µm^2^)). Source data

**Extended Data Fig. 1:**
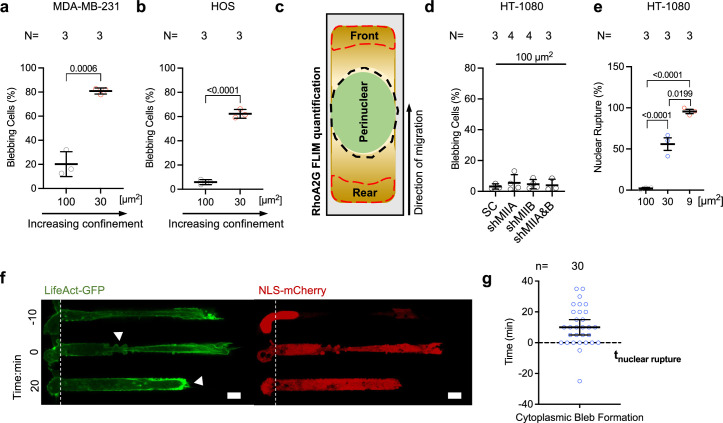
Confinement induces nuclear envelope ruptures and cell blebbing. **(a, b)** Percentage of MDA-MB-231 (**a**) or HOS (**b**) cells migrating with a blebbing phenotype in moderately-confining (100-µm^2^) and confining (30-µm^2^) channels (n ≥ 20 cells per experiment from N = 3 experiments). **(c)** Schematic illustrating the regions quantified for RhoA activity via FLIM in HT-1080 cells migrating in microchannels. **(d)** Percentage of scramble control, MIIA-, MIIB-, or MIIA and IIB- knockdown HT-1080 cells migrating with a blebbing phenotype in 100-µm^2^ channels (n ≥ 15 cells per experiment from N experiments). **(e)** Percentage of HT-1080 cells that experienced NE rupture in 100-µm^2^, 30-µm^2^, and 9-µm^2^ channels, as evaluated by NLS-mCherry exit into the cytoplasm (n ≥ 10 cells per experiment from N = 3 experiments). **(f)** Image sequence (confocal slice) of representative HT-1080 cells experiencing NE ruptures, as assessed by NLS-mCherry localization, and subsequently forming cytoplasmic blebs, as assessed by LifeAct-GFP, in confining (30-µm^2^) channel. White dashed lines indicate channel entrance. White arrowheads indicate membrane blebs. Scale bars: 10 µm. **(g)** Time required for the initial formation of cytoplasmic blebs versus the first NE rupture in confining (30-µm^2^) channels, as assessed by cells expressing NLS-mCherry and LifeAct-GFP (n = 30 cells from N = 3 experiments). Values represent mean±S.D. (**a**,**b**,**d**,**e**,**g**). Statistical significance was assessed by unpaired t-test (**a**,**b**), or one-way ANOVA followed by Tukey’s multiple comparisons test after log transformation (**e**). Source data

To delineate the effect of increasing confinement on the cell migration mode and efficiency, we induced HT-1080 fibrosarcoma cells to migrate through moderately confining (*A* = 100 µm^2^, *W* (width) *=* 10 µm, *H* (height) = 10 µm), confining in the dorsoventral direction (*A* = 30 µm^2^, *W* = 10 µm, *H* = 3 µm), or tightly confining (*A* = 9 µm^2^, *W* = 3 µm, *H* = 3 µm) collagen I-coated channels. Most (~85%) of the HT-1080 cells in moderately confining channels exhibited finger-like protrusions consistent with their characteristic mesenchymal phenotype (Fig. 1a,b). As the degree of confinement increased, cells switched from a primarily mesenchymal to a bleb-based migration mode (Fig. 1a,b). Blebbing cells displayed a pill-like morphology with plasma membrane blebs, which were identified as sphere-like bulges (Fig. 1a). Confinement-induced cell blebbing was also observed for MDA-MB-231 breast cancer cells and human osteosarcoma (HOS) cells (Extended Data Fig. 1a,b).

Because bleb formation requires the activation of RhoA/myosin II-dependent contractility^8,11^, we used confocal fluorescence-lifetime imaging microscopy (FLIM) coupled with a Förster resonance energy transfer (FRET)-based RhoA2G activity biosensor^17,21^ to quantify the RhoA activity. In line with an increase in the blebbing phenotype, cells migrating in confining or tightly confining channels displayed increased RhoA activity, as evidenced by the decreased donor fluorescence lifetimes compared with cells in moderately confining channels (Fig. 1c,d). RhoA activity was polarized along the long axis of the migrating cells, with maximum levels detected in areas of membrane blebs at the leading and trailing edges of cells in confining and tightly confining microchannels (Fig. 1c,e and Extended Data Fig. 1c). By contrast, cells in moderately confining channels displayed relatively uniform basal levels of RhoA activity (Fig. 1e). Knockdown of *myosin IIA* (MIIA or MYH9)^17^ converted cells to a predominantly mesenchymal phenotype in tightly confining channels (Fig. 1f) without affecting the migration mode in moderately confining channels (Extended Data Fig. 1d)^11^. The depletion of *myosin IIB* (MIIB or MYH10)^17^ had no effect on the migration phenotype in tight confinement (Fig. 1f). Although the inhibition of the RhoA/ROCK pathway with Y27632 (10 μM) suppressed the extent of cell entry in all channels, it delayed this process more pronouncedly in confining and tightly confining channels compared with moderately confining ones (Fig. 1g,h). These results reveal that RhoA/ROCK/MIIA-dependent contractility, which converts cells to a bleb-based migration phenotype, becomes elevated in confinement and facilitates cell entry into confined spaces.

In addition to plasma membrane blebbing, confinement—by exerting mechanical stress on the nucleus—promoted nuclear blebbing, which frequently resulted in NE rupture (Extended Data Fig. 1e) and the exchange of contents between the nucleus and the cytoplasm^17,22,23^. In line with their elongated morphology on two dimensions, HT-1080 cells initiated entry into polydimethylsiloxane (PDMS)-based confining channels and displayed a mesenchymal migration mode, and the majority transitioned on channel entry to a bleb-based migration phenotype (Fig. 1b and Extended Data Fig. 1f). The transition of HT-1080 cells from mesenchymal to cytoplasmic blebbing phenotype in confining (30-µm^2^) channels frequently coincided with or followed NE rupture events (Extended Data Fig. 1f,g). This coincidence of confinement-induced NE rupture and cell blebbing suggested that nuclear constituents escaping to the cytoplasm following NE rupture could promote RhoA/myosin II activation, thereby facilitating cell migration in confining spaces. We noted that the elevation of RhoA activity at the front and rear cell edges (Fig. 1c,e), the involvement of Rho/ROCK-mediated myosin II activation (Fig. 1f–h) and the exchange of nucleocytoplasmic materials on NE rupture (Extended Data Fig. 1f) observed in confinement are reminiscent of cytokinesis^16^, which involves the breakdown of NE and the subsequent assembly of the contractile ring at the cleavage furrow. These similarities prompted us to examine the potential role of cytokinesis regulators in confined migration.

## Confinement promotes anillin accumulation at the cell poles

**Fig. 2:**
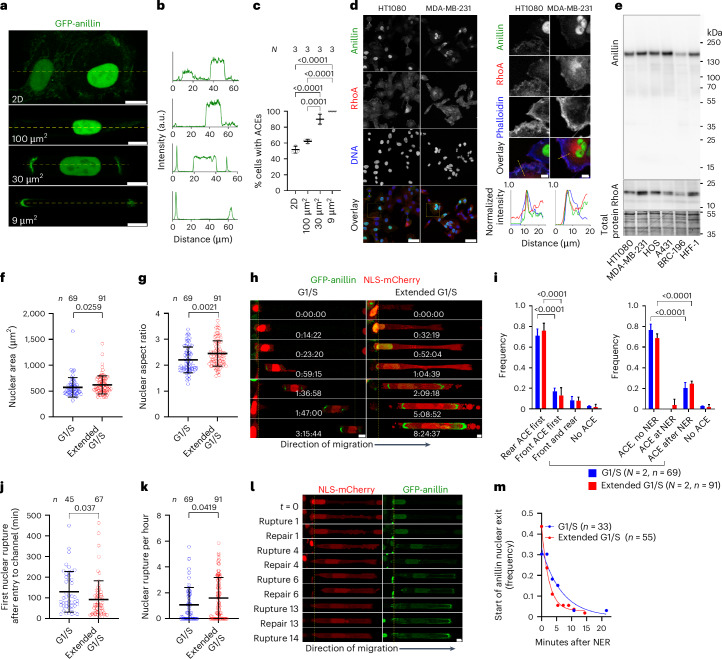
ACEs exist in cells on two dimensions and are amplified by confinement. **a**–**c**, Live-cell images (**a**), linescans of GFP-anillin intensity (**b**) and quantification of ACE frequency (**c**) in GFP-anillin-expressing HT-1080 cells in collagen I-coated PDMS channels from *N* = 3 experiments. Scale bars, 10 µm. **d**, Presence of endogenous ACEs in wild-type HT-1080 and MDA-MB-231 on 2D substrates as shown by immunofluorescence staining. Scale bars, 50 µm. Subcortical anillin colocalization with RhoA and actin is shown. Scale bars, 10 µm (inset). The insets show the quantification of 10-pixel-wide linescans (dashed lines) of anillin, RhoA and actin, showing colocalization at the plasma membrane. **e**, Representative western blot showing anillin and RhoA expression levels in different cell types. **f**,**g**, Nuclear area (**f**) and nuclear aspect ratio (**g**) of early and extended G1/S cells (*n* cells from *N* = 2 experiments). **h**, Anillin nuclear exit occurs either concurrently with or after NE ruptures. Early and extended G1/S cells form ACEs at their trailing edge during cell entry into confinement before NE rupture. ACEs are further enriched after rupture. Time lapse taken at 108-s intervals. The yellow dashes indicate the microchannel entrance. Numbers indicate the time from start of channel entry (hh:mm:ss). **i**, Frequency of ACE formation at the rear and/or front of early and extended G1/S cells during channel entry and frequency of ACE formation before, at or after NE rupture (NER) events in synchronized cells (*n* cells from *N* = 2 experiments). **j**,**k**, Comparisons of timing of first NE rupture following cell entry into confinement (**j**) and hourly rate of NE ruptures (**k**) between early and extended G1/S phase cells (*n* cells from *N* = 2 experiments). **l**, Images of an extended G1/S cell experiencing repeated NE ruptures, which resulted in intensifying ACEs. Scale bars, 10 µm. **m**, Timing of anillin escape to the cytoplasm following the first NE rupture (*n* cells from *N* = 2 experiments). All images were obtained by confocal microscopy. Values represent the mean ± s.d. Statistical analysis was performed by one-way ANOVA followed by Tukey’s multiple comparisons (**c**), two-tailed unpaired *t*-test (**g**) after log transformation (**j**), two-tailed Mann–Whitney test (**f** and **k**) or two-way ANOVA followed by Dunnett’s post hoc test (**i**). Source data

**Extended Data Fig. 2:**
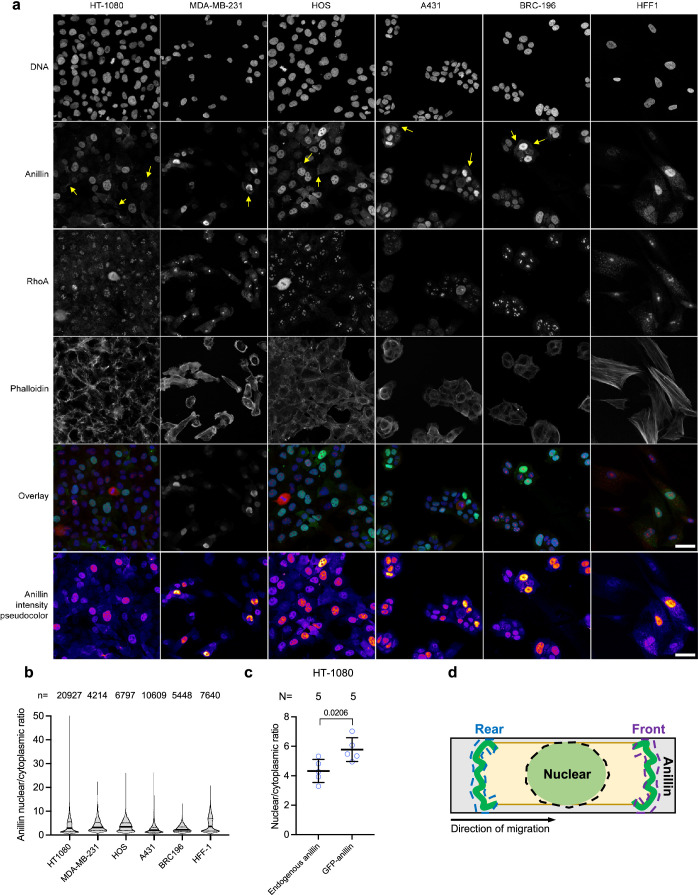

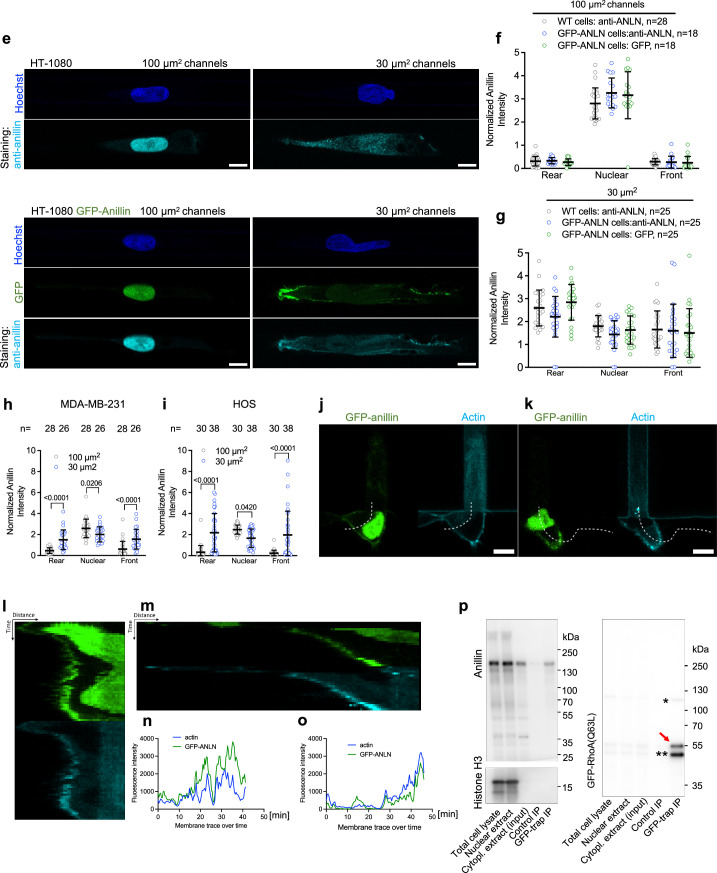

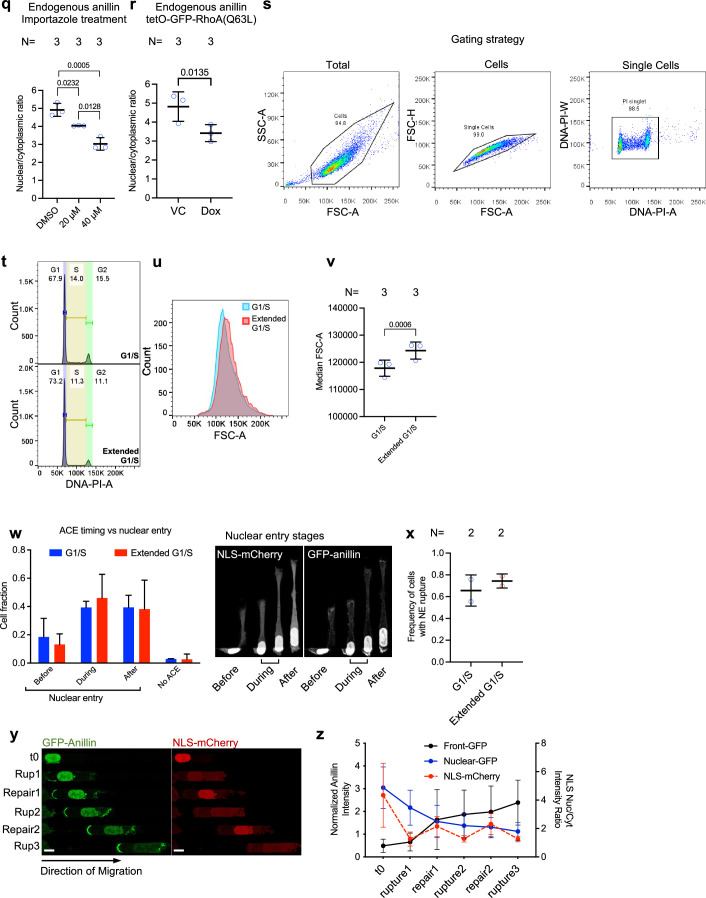
Cytoplasmic anillin is detected in different cell types and becomes enriched upon NE rupture in confinement. **(a,b)** Cytoplasmic anillin exists in numerous cell lines: HT-1080, MDA-MB-231, HOS, A431, BRC-196 and HFF-1, with ACEs indicated by yellow arrows, as assessed by immunofluorescence staining (**a**) and quantification of nuclear-to- cytoplasmic intensity of anillin (**b**) (n cells from N = 3 experiments). Scale bars: 10 μm. **(c)** Quantification of nuclear-to-cytoplasmic ratio of endogenous anillin and GFP-anillin (n ≥ 499 cells per experiment from N = 5 experiments)**. (d)** Schematic depicting the cell front, rear, and nuclear cell regions as used for quantification of anillin. **(e)** Representative images of HT-1080 WT or HT-1080 GFP-anillin (WT) cells migrating in moderately-confining or confining channels showing that GFP-anillin localization correlates with that of total anillin, as assessed by GFP-anillin (green) imaging and confirmed by immunofluorescence staining with Hoechst 33342 (blue) and anti-anillin antibody (cyan). Scale bars: 10 µm. **(f,g)** Quantification of localization of GFP-anillin and total anillin in HT-1080 WT and HT-1080 GFP-anillin (WT) cells migrating in 100 µm^2^ (n cells from N = 2 experiments) (**f**) or 30 µm^2^ channels (n cells from N = 2 experiments) (**g**). **(h,i)** Quantification of GFP-anillin localization in MDA-MB-231 (n cells from N = 3 experiments) (**h**) or HOS (n cells from N = 3 experiments) (**i**) cells migrating in 100 µm^2^ and 30 µm^2^ channels. **(j,k)** Representative images of two HT-1080 cells expressing GFP-anillin and labelled with SPY650-FastAct showing co-localization of GFP-anillin (green) and actin (cyan) during cell entry into confining microchannels. Scale bars: 10 μm. **(l,m)** Kymographs of GFP-anillin (green) and actin (cyan) along the curved linescans, which tracked rear plasma membrane retractions during cell entry, from (**j**) and (**k**), respectively (dashed white lines). **(n,o)** Fluorescence intensity profiles of GFP-anillin and actin on the plasma membrane over time, obtained by linescanning the edges of the kymographs in (**l**) and (**m**), respectively, showing co-fluctuation of GFP-anillin and actin signal along the cell membrane. The image intensity was uniformly adjusted in all images for optimal visualization of anillin and actin on the cell membrane. (**p**) Representative co-immunoprecipitation showing RhoA and anillin interaction in the cytoplasm, as indicated by presence of anillin in GFP-pulldown of lysates from HT-1080 cells expressing the constitutively active RhoA construct GFP-RhoA(Q63L) (left blot). Control detection is shown in the right blot, demonstrating the concentration of GFP-RhoA by the GFP-trap IP compared to nuclear and cytoplasmic lysate extracts. Red arrow: expected GFP-RhoA band (slow-migrating form). *: Non-specific band. **: Fast-migrating form of RhoA, likely directly interacting with anillin^3^. **(q)** Dose-dependent effects of importin-β inhibitor importazole on nuclear/cytoplasmic ratios of endogenous anillin in HT-1080 cells plated on 2D substrates (n ≥ 899 cells per experiment from N = 3 experiments). **(r)** Overexpression of constitutively active RhoA with the doxycycline-inducible RhoA(Q63L) mutant leads to higher anillin cytoplasmic retention for cells on 2D substrates (n ≥ 951 cells per experiment from N = 3 experiments). **(s,t)** Flow cytometry gating strategy example (**s**) and representative FACS analysis (**t**) with propidium-iodide staining showed extended hydroxyurea treatment (HU, bottom graph) stalled cells in G1/S phase compared to non-treated control (top graph). **(u,v)** Cells in the extended G1/S phase, compared to those in the early G1/S phase, display a larger size, as evidenced by the FSC-A shift from a representative FACS analysis (**u**) and median FSC-A values (n ≥ 10,000 cells per experiment from N = 3 experiments) (**v**). **(w)** Quantification of frequency of cells with no ACEs or ACEs forming before, during, or after nuclear entry into confining channels. Representative images of nuclear entry stages on the right (n ≥ 69 cells from N = 2 experiments). **(x)** Comparison of NE rupture frequency between early and extended G1/S phase cells (n ≥ 69 cells from N = 2 experiments). **(y)** Representative images of an unsynchronized cell experiencing repeated NE ruptures which resulted in intensifying ACEs. **(z)** Quantification of GFP-anillin intensity at the cell front and in the nucleus as well as NLS-mCherry nuclear-to-cytoplasmic intensity of unsynchronized cells in confinement (n = 16 cells from N = 2 experiments). Values represent mean±S.D. All representative images are from a single confocal slice. Statistical significance was assessed by unpaired t-test (**c**), or two-way ANOVA followed by Sidak’s test (**h**,**i**), one-way ANOVA followed by Tukey’s multiple comparisons test (**q**), or paired t-test (**r**,**v**). Source data

The assembly of the actomyosin-based contractile ring during cytokinesis critically depends on the activation of RhoA^16^ by RhoGEF Ect2, which is anchored to the equatorial cortex via the scaffold protein anillin, in a manner that could involve Ect2–plasma membrane binding via its PH domain^15^. Although anillin and Ect2 are predominantly nuclear during interphase^14,24^, anillin accumulates at cell–cell junctions in epithelial MCF7 cell monolayers^25^, and increased cytoplasmic-to-nuclear Ect2 levels are observed in human colorectal cancer tumours compared with normal tissues^19^. Live-cell imaging of GFP-anillin-expressing HT-1080 cells revealed its predominant nuclear localization accompanied by varying degrees of diffuse cytoplasmic signal and accumulation at the plasma membrane when grown on 2D surfaces (Fig. 2a,b). Both nuclear and cytoplasmic endogenous anillin were also observed by immunofluorescence in HT-1080 and five additional tissue culture cell types, including MDA-MB-231 triple-negative breast cancer cells and A431 epidermoid carcinoma cells (Extended Data Fig. 2a,b). Although GFP-anillin expressed in HT-1080 cells was moderately more nuclear compared with endogenous anillin probably due to the added GFP tag size (Extended Data Fig. 2c), these results suggest that GFP-anillin mimics the localization pattern of the endogenous anillin.

Although most HT-1080 cells migrating in moderately confining channels exhibited a similar anillin nuclear-cytoplasmic localization pattern as cells on 2D substrates, higher degrees of confinement (30 µm^2^ and 9 µm^2^) sharply increased the frequency of cells with ACEs within narrow bar- or arch-like areas having lengths of 2–3 µm (Fig. 2a–c and Extended Data Fig. 2d), as determined by live-cell imaging and immunofluorescence (Extended Data Fig. 2d–g). Similar localization patterns were also detected with MDA-MB-231 and HOS cells in moderately confining and confining channels (Extended Data Fig. 2h,i).

As previously reported^16,25^, anillin colocalizes with filamentous actin and RhoA at these cytoplasmic sites of accumulation (Fig. 2d) irrespective of the varying cell-type-specific anillin and RhoA protein expression levels (Fig. 2e). In line with these findings, live-cell imaging using GFP-anillin HT-1080 cells labelled with SPY650-FastAct reveals the colocalization and concurrent accumulation of anillin and filamentous actin at the plasma membrane, particularly at the cell trailing and leading edges during cell entry into confining spaces (Extended Data Fig. 2j–o and Supplementary Videos 1–4). Moreover, coimmunoprecipitation experiments showed that the constitutively active form of RhoA (RhoA Q63L) readily interacts with anillin in cytoplasmic extracts of interphase HT-1080 cells (Extended Data Fig. 2p), demonstrating that the anillin-RhoA-GTP scaffolding function is not limited to mitotic cells during cytokinesis^16^ or adherent junctions of epithelial cell monolayers^25^. Combining these findings with the absence of a documented nuclear export signal on anillin, the control of the anillin nucleocytoplasmic distribution presumably involves a balance between its active nuclear import and cytoplasmic retention at the subcortical actomyosin meshwork and its direct binding to the plasma membrane via its PH domain^25^. Anillin possesses two distinct nuclear localization signal (NLS) sequences^26^ recognized by transportin 1 (TNPO1/KPNB2)^24^ and importin-β (KPNB1) in complex with its adaptors of the importin α family^26^. Treatment with importazole, an importin-β inhibitor that does not affect TNPO1/KPNPB2-dependent nuclear import^27^, reduced the nuclear accumulation of endogenous anillin in HT-1080 cells, as evaluated by immunofluorescence (Extended Data Fig. 2q), indicating that Ran-importin-β/α-mediated nuclear import has an essential role in maintaining anillin nuclear localization during interphase. In particular, the doxycycline-induced expression of constitutively active GFP-RhoA-Q63L reduced the nuclear localization of endogenous anillin, presumably due to the increased cytoplasmic retention of anillin at RhoA-GTP-amplified cytoplasmic actomyosin (Extended Data Fig. 2r).

Considering the critical role of the nucleus as a mechanosensor^28^, we next examined the effects of nuclear size on anillin localization during confined cell migration. Given that the nuclear size generally scales with the cell size, which typically increases as cells approach mitosis in many cell types^29^, we prepared populations of HT-1080 cells expressing GFP-anillin and NLS-mCherry that were enriched in early versus extended G1/S phase via serum withdrawal or serum withdrawal followed by hydroxyurea treatment, respectively. Because exposure to serum-provided growth factors should enable cell growth but hydroxyurea causes arrest at the G1/S transition^30^, we expected that extended G1/S arrest could increase the nuclear size of these early interphase cells. Indeed, measurements in live cells entering the confining channels showed a significantly increased nuclear size and aspect ratio in the extended G1/S population, as quantified by nuclear morphometric analysis of maximum-intensity *z* projections (Fig. 2f,g). Fluorescence-activated cell sorting analysis confirmed the G1/S cell state of both cell populations (Extended Data Fig. 2s,t) and the effect of extended G1/S arrest on increased cell volume (Extended Data Fig. 2u,v), corroborating our data on nuclear size (Fig. 2f,g).

Confocal live-cell imaging revealed that entry into confining channels for both early and extended G1/S phase cell populations was frequently marked by the accumulation of GFP-anillin within ACEs predominantly at the trailing cell edges (Fig. 2h,i). Although the formation of these spatially localized GFP-anillin-rich zones was dynamic over time and varied from cell to cell, several prevailing patterns were observed. First, irrespective of the cell treatment and the prevailing nuclear size, ACEs were induced by cell entry into confinement in >97% of cells (Fig. 2i). Moreover, ACEs formed during or shortly after nuclear entry into confining channels in >80% of the cells, suggesting nuclear confinement as a trigger of ACE formation (Extended Data Fig. 2w and Supplementary Video 5). Although the frequency of cells with at least one NE rupture was similar in both populations (Extended Data Fig. 2x), cells in the extended G1/S phase experienced the first NE rupture at an earlier timepoint and underwent NE ruptures at a higher rate compared with early G1/S cells in confinement (Fig. 2j,k), presumably due to their larger nuclear size and elevated nuclear compression and stretching in confinement (Fig. 2f,g). These findings highlight the critical roles of the nucleus and its size in confined migration.

ACEs predominantly formed either before or in the absence of NE rupture in both early and extended G1/S-phase cell populations (Fig. 2i), as determined by the concurrent monitoring of nuclear-to-cytoplasmic NLS-mCherry ratio (Fig. 2h,i), indicating that nuclear confinement triggers ACE formation by anillin recruitment from pre-existing cytoplasmic pools. We next examined the timing of GFP-anillin nucleocytoplasmic relocalization after confinement-induced NE rupture identified by the abrupt decrease in the nuclear-to-cytoplasmic NLS-mCherry ratio (Fig. 2l). Quantification of the subcellular distribution of GFP-anillin revealed that although the release of nuclear GFP-anillin to the cytoplasm coincided with NE rupture in ~40% of confined cells, anillin nuclear exit was delayed by up to 20 min in the rest of the cells (Fig. 2m). These data suggest that the retention of anillin at the nuclear structures—that remain to be identified—slows its cytoplasmic escape on the breach of NE integrity, and contributes to controlling its nuclear-cytoplasmic distribution. Live-cell imaging showed that NE rupture promoted anillin enrichment at the cell leading and trailing edges, accompanied by a decreased nuclear-to-cytoplasmic GFP-anillin ratio for both G1/S-synchronized (Fig. 2h,l and Supplementary Video 6) and unsynchronized cells (Extended Data Fig. 2y,z), suggesting that anillin released from the nucleus preferentially accumulated within the ACEs. Taken together, cytoplasmic anillin is recruited to the cell front and rear edges during nuclear entry into confining microchannels, and its accumulation in these locations is further enhanced due to confinement-induced NE rupture.

## Stiffness and pore size regulate anillin localization

**Fig. 3:**
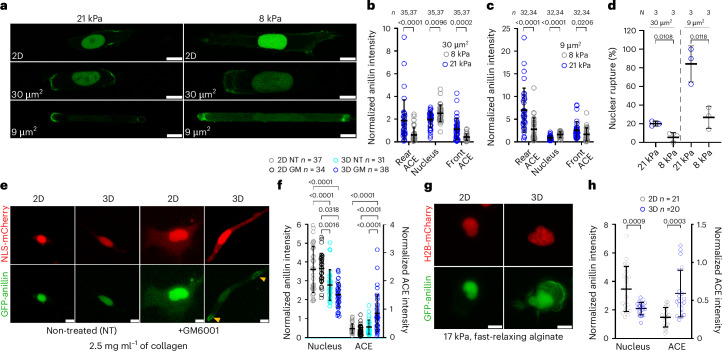
Migration in biomimetic 3D environments promotes anillin recruitment to ACEs. **a**, Representative images of GFP-anillin localization in HT-1080 cells on 2D gels and inside 30-µm^2^ and 9-µm^2^ collagen I-coated channels of 21- or 8-kPa stiffness. Scale bars, 10 µm. **b**,**c**, Quantification of GFP-anillin intensity in the rear, nuclear and front cell regions of cells inside compliant (21 or 8 kPa) channels with cross-sectional area of 30 µm^2^ (**b**; *n* cells from *N* = 4 experiments) or 9 µm^2^ (**c**; *n* cells from *N* = 4 experiments). **d**, NE rupture frequency in cells inside compliant (21 kPa versus 8 kPa) 30-µm^2^ and 9-µm^2^ channels (*N* = 3 experiments). **e**, Representative images of GFP-anillin and NLS-mCherry localization in HT-1080 cells on 2D or 3D collagen gels with or without the MMP inhibitor GM6001. The arrowheads indicate GFP-anillin at the cell poles. Scale bars, 10 µm. **f**, Quantification of GFP-anillin intensity in the nucleus and within ACEs on 2D or 3D collagen gels with or without GM6001 (*n* cells from *N* = 5 experiments). **g**, Representative images of GFP-anillin and H2B-mCherry localization in HT-1080 cells on 2D or 3D viscoelastic alginate gels. Scale bars, 10 µm. **h**, Quantification of GFP-anillin intensity in the nucleus or within ACEs in cells on 2D or within 3D alginate gels (*n* cells from *N* = 2 experiments). Confocal images are either from a single slice (**a**) or are the maximum-intensity *z* projections (**e** and **g**). Statistical significance was assessed by a two-tailed Mann–Whitney test (**b** and **c**), two-tailed unpaired *t*-test (**d** and **h**) or Kruskal–Wallis followed by Dunn’s multiple comparisons (**f** (nuclear anillin)) or one-way ANOVA followed by Tukey’s multiple comparisons test after log transformation (**f** (ACE intensity)). Source data

We sought to extend our findings from stiff PDMS-based confining microchannels to other physiologically relevant microenvironments. First, we examined the subcellular distribution of anillin in a hydrogel-encapsulated microchannel array (HEMICA), which enables precise control over channel stiffness and size^31^. The HEMICA device consisted of an array of four-walled, compliant polyacrylamide-based channels, which were either confining (*A* ≈ 30 µm^2^, *W* ≈ 10 µm, *H* ≈ 3 µm) or tightly confining (*A* ≈ 9 µm^2^, *W* ≈ *H* ≈ 3 µm) to recapitulate the dimensions of the PDMS devices. The channels were fabricated with stiffness values of 8 kPa or 21 kPa to emulate (patho)physiologically relevant conditions^32^. In line with data using PDMS channels, anillin was primarily localized in the nucleus of cells on 2D HEMICA surfaces irrespective of the substrate stiffness (Fig. 3a), but became increasingly polarized, forming ACEs as the stiffness and degree of confinement increased (Fig. 3b,c). In particular, cells inside stiffer (21 kPa) confining channels displayed a higher accumulation of anillin at both cell front and rear compared with cells inside softer (8 kPa) ones (Fig. 3b), whereas in tight confinement, a more intense anillin signal was only detected at the cell rear (Fig. 3c). The higher anillin accumulation in migrating cells inside stiffer than softer channels is attributed to the higher rate of NE rupture (Fig. 3d), which promotes anillin escape from the nucleus and ACE formation via actomyosin binding.

**Extended Data Fig. 3:**
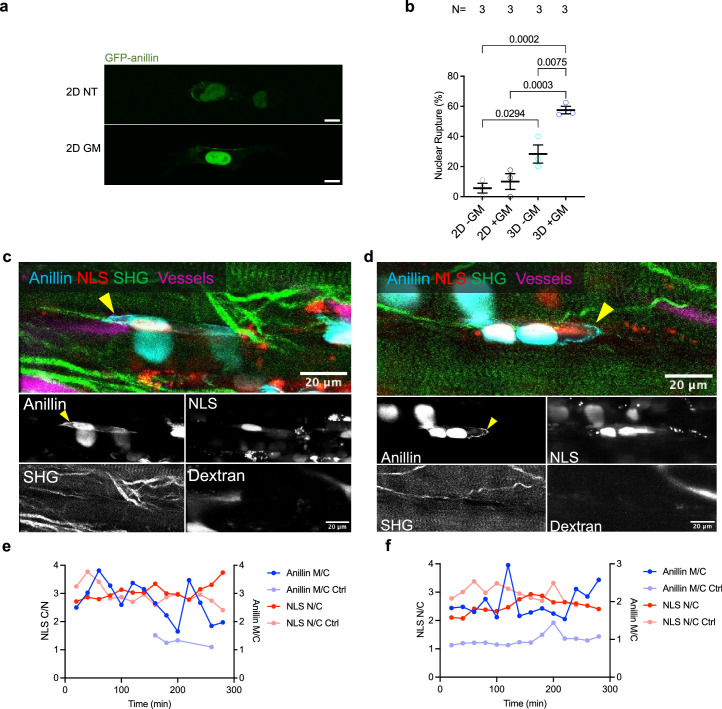
Cytoplasmic anillin is detected in 2D and 3D hydrogels *in vitro* and in invading tumor cells *in vivo.* **(a)** Representative images (confocal slice) of HT-1080 cells expressing GFP-anillin with ACEs on 2D collagen gels with or without GM6001. **(b)** Percentage of HT-1080 cells experiencing NE rupture events in 2D or 3D collagen gels in the presence or absence of GM6001, as assessed by NLS-mCherry localization (n ≥ 5 cells per experiment from N = 3 experiments). **(c,d)** Persisting anillin localization at the membrane (yellow arrowheads) monitored by multiphoton microscopy (maximum z-projection). Scale bars: 20 µm. **(e,f)** Quantification of NLS-mCherry nuclear/cytoplasmic ratio and anillin membrane/cytoplasmic ratio over time showing persistent ACEs (**e**) or fluctuating accumulation of ACEs (**f**) even without detectable NE ruptures. Neighboring cells that showed no ACEs and NE ruptures were quantified as controls (Ctrl). Values represent the mean±S.D. Statistical significance was assessed by one-way ANOVA followed by Tukey’s multiple comparisons test. Source data

Next, we examined anillin localization in a 3D ECM environment, which recapitulates the stiffness and porosity of in vivo tissues^33^. Pronounced NE rupture has previously been observed in cells embedded in 3D collagen I gels following matrix metalloproteinase (MMP) inhibition due to the inability of cells to widen pores via the enzymatic cleavage of collagen fibres^22^. We verified this finding using NLS-mCherry-tagged cells embedded in 2.5 mg ml^–1^ collagen I gels (Fig. 3e). Given that NE rupture amplifies the formation of ACEs in confining channels (Extended Data Fig. 2y,z), we predicted an increased cytoplasmic anillin accumulation in 3D collagen gels especially after MMP inhibition. Indeed, although GFP-anillin was mainly nuclear on 2D collagen I gels with some presence in the cell periphery similar to cells on 2D glass (Fig. 3e,f and Extended Data Fig. 3a), the GFP intensity within ACEs was more than twofold higher in MMP-inhibited cells migrating through 3D gels (Fig. 3e,f). Of note, in the absence of MMP inhibition, the nuclear intensity of GFP-anillin was significantly lower in 3D than on 2D gels (Fig. 3f, left *y* axis), suggesting that the moderately increased NE rupture frequency in three dimensions (Extended Data Fig. 3b) probably contributed to the decrease in nuclear anillin levels, although it was not sufficient to detectably increase the ACE formation.

Although collagen gels recapitulate some aspects of the physiological tissue microenvironment, natural ECM and living tissues exhibit viscoelastic behaviours, often displaying stress relaxation over different characteristic timescales (*t*_1/2_; ref. ^34^). To extend our findings to matrices of physiologically relevant viscoelastic properties, we examined anillin localization in fast-relaxing (*t*_1/2_ = 1 min) alginate gels with a stiffness of 17 kPa. GFP-anillin was primarily localized to the nucleus of cells on 2D alginate gels (Fig. 3g). By contrast, the nuclear localization of anillin decreased significantly in 3D alginate gels concomitant with an almost twofold increase in the ACE intensity (Fig. 3g,h), indicating that the physiologically relevant fast-relaxing 3D alginate environment promotes anillin localization at the cell edges.

**Fig. 4:**
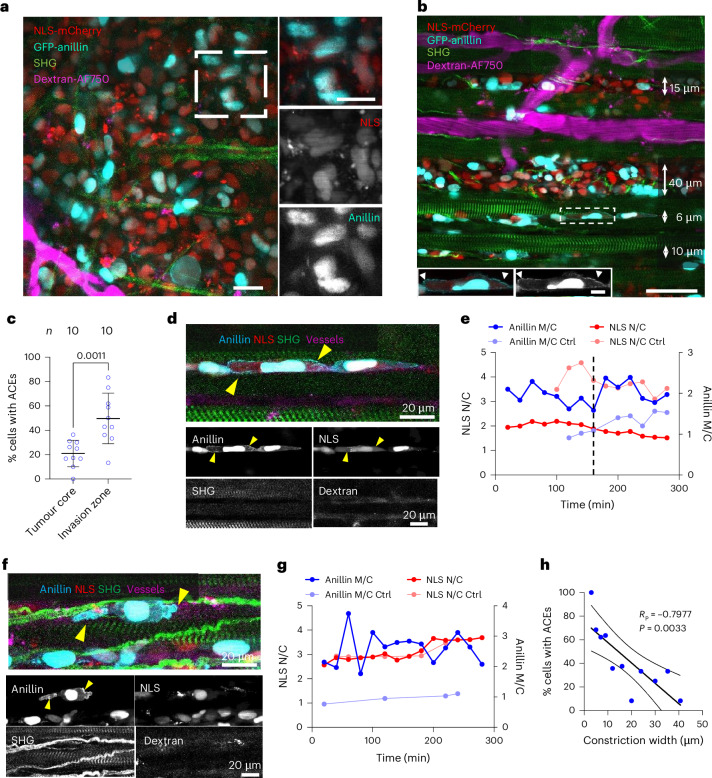
Confinement enhances anillin nuclear exit and accumulation at the plasma membrane in vivo*.* **a**, HT-1080 GFP-anillin/NLS-mCherry tumour xenografts invading the mouse dermis were monitored by intravital multiphoton microscopy through a dorsal skin-fold chamber. Images represent an overview and details of the tumour core obtained 2 days after tumour implantation 50 µm below the tumour surface. NLS-mCherry (red), GFP-anillin (cyan), SHG-positive collagen fibres (green), Alexa Fluor 750 (AF750)-positive blood vessels and fluorescence-positive phagocytes after dextran uptake (magenta). Scale bars, 20 µm. **b**, Single-cell and multicellular invasion along interstitial clefts between blood vessels, myofibers and interstitial collagen networks of different widths. The double-headed arrows indicate the average width of each tissue track after the entry of tumour cells. An example cell in the zoomed-in images show GFP-anillin (cyan or greyscale) redistributed to the cell edge (arrowheads in the zoomed-in insets) migrating between two myofibers. Scale bar, 50 µm (overview); 5 µm (inset). **c**, Percentage of cells with ACEs in the invasion zone versus the tumour core. Data are represented as the mean percentage per region, calculated from ten regions in the tumour core (206 cells) and ten invasion zones (204 cells) pooled from three mice. **d**,**f**, Representative images of GFP-anillin/NLS-mCherry-expressing HT-1080 cells migrating between myofibers in mouse deep dermis display a strong formation of ACEs (yellow arrowheads) in the presence (**d**) or absence (**f**) of NE ruptures, as revealed by the intravital microscopy of GFP-anillin, NLS-mCherry, collagen and myofibers (SHG), and blood vessels (dextran). **e**,**g**, Quantification of NLS-mCherry nuclear/cytoplasmic ratio (NLS N/C) and GFP-anillin membrane/cytoplasmic ratio (anillin M/C) over time for **d** and **f**, respectively, which shows strong ACE formation in the presence (**e**; dashed line) or absence (**g**) of NE ruptures, as revealed by the high membrane/cytoplasmic ratios of anillin intensity (M/C ≥ 2) compared with nearby control cells that did not possess such ACEs (Ctrl). Scale bars, 20 µm. **h**, Inverse correlation between the percentage of cells displaying cytoplasmic anillin and the width of interstitial space. The images are the maximum-intensity *z* projections from multiphoton microscopy. Data represent *n* = 195 cells obtained from ten invasion zones pooled from three mice. Values are represented as mean ± s.d. Statistical significance was assessed by a two-tailed unpaired *t*-test (**c**) and Pearson correlation (**h**). Source data

Finally, we examined the localization pattern of anillin in cells migrating in vivo. To this end, we implanted HT-1080 cells expressing GFP-anillin and NLS-mCherry into the deep dermis of nude mice bearing an optical imaging window for in vivo monitoring in real time^35^. Tumour cell invasion into the collagen-rich interstitial tissue was longitudinally monitored 4–11 days post-implantation; 3D tissue constituents, including fibrillar collagen and myofibers (second-harmonic generation (SHG)), macrophages and blood vessels (70-kD dextran-Alexa Fluor 750), were corecorded alongside monitoring the localization of GFP-anillin in the nucleus and cytoplasm. Although the majority of non-invading cells at the tumour boundary retained primarily nuclear anillin localization (Fig. 4a), the accumulation of GFP-anillin at the plasma membrane was increased in invading cells (Fig. 4b,c), supporting a pro-invasive role for such anillin localization in vivo. Although the initiation of NLS-mCherry cytoplasmic leakage correlated with anillin enrichment in the cytoplasm and at the plasma membrane (Fig. 4d,e and Supplementary Video 7), it also occurred independently of NE rupture (Fig. 4f,g, Extended Data Fig. 3c–f and Supplementary Videos 8–10), presumably due to the cytoplasmic redistribution of anillin as observed in biomimetic in vitro models (Fig. 2h,i and Supplementary Videos 3 and 5). Consistent with a mechanoresponse, and similar to microfluidic assays (Figs. 2c and 3b,c), the percentage of cells displaying cytoplasmic anillin localization increased with an increasing confinement in vivo, reaching almost 100% in narrow perimuscular tissue clefts (Fig. 4h). These confining (<10 µm) tracks in vivo hinder the multicellular arrangement typical of collective invasion, and instead enforce chain-like single-cell migration, with bilateral cell contact to comparably stiff structures of the dermis, including collagen bundles and myofibers (5 kPa to >100 kPa)^32,36^. Conversely, in wider channels, invading cells are less confined, as they interact with both tissue structures and much softer bodies of neighbour cells (<2 kPa)^37^. Thus, in accordance with in vitro findings, tissue stiffness and confinement in vivo probably generate cooperating mechanochemical triggers in inducing and sustaining anillin translocation to the membrane. In contrast to in vitro observations, the frequency of membrane blebbing in the mouse dermis was not as pronounced even though anillin was still detected in the cytoplasm, although at lower levels. This might be attributed to elevated levels of Rac activators in the tumour microenvironment in vivo due to cytokine and growth factor release^38,39^, which may lead to the coexistence of cortical contractility and filamentous protrusions.

## Anillin and Ect2 drive myosin II-dependent bleb-based motility

**Fig. 5:**
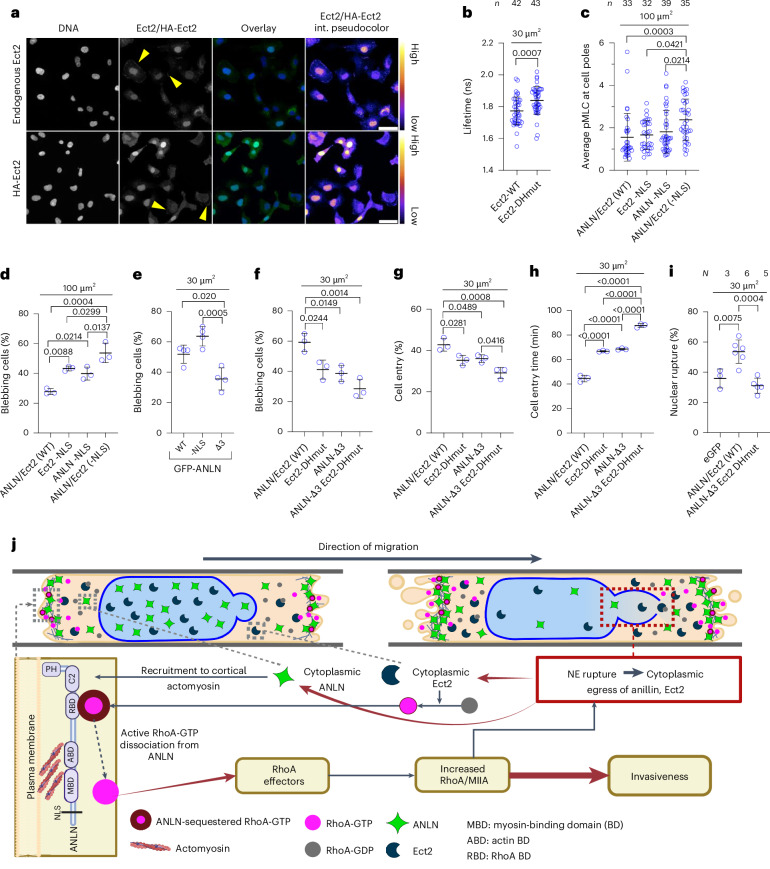
Cytoplasmic anillin and Ect2 enhance RhoA/myosin II-dependent contractility and bleb-based confined cell migration. **a**, Representative images (single confocal sections) of HT-1080 cells on 2D substrates labelled for endogenous or HA-tagged Ect2, showing the presence of Ect2 in both nucleus and cell cytoplasm. The yellow arrowheads indicate endogenous Ect2 at the plasma membrane. Scale bars, 50 µm. **b**, Donor fluorescence lifetime of RhoA activity biosensor for HT-1080 cells expressing HA-Ect2 or HA-Ect2 with mutations in its DH domain in confining channels, as measured by FLIM–FRET (*n* cells from *N* = 3 experiments). **c**, Average pMLC intensity at the cell poles relative to the cytoplasmic pMLC intensity excluding the poles, as assessed from fixed and stained HT-1080 cells expressing either both GFP-anillin and HA-Ect2 (ANLN/Ect2 (WT)), or GFP-anillin or HA-Ect2 with a mutated NLS domain (ANLN-NLS or Ect2-NLS), or both GFP-anillin and HA-Ect2 with mutated NLS domains in moderately confining channels (*n* cells from *N* = 3 experiments). **d**, Percentage of blebbing cells in moderately confining channels for HT-1080 cells expressing the constructs in **c** (*n* ≥ 47 cells per experiment from *N* = 3 experiments). **e**, Percentage of blebbing cells in confining channels for HT-1080 cells expressing GFP-anillin (WT); GFP-anillin with deletion in its NLS domain (-NLS); or deletion in its NLS-, myosin- and actin-binding domains (Δ3; *n* ≥ 10 cells per experiment from *N* = 4 experiments). **f**, Percentage of blebbing cells in confining channels for HT-1080 expressing both GFP-anillin/HA-Ect2 (ANLN/Ect2 (WT)); HA-Ect2-DHmut; GFP-anillin with NLS, myosin and actin deletion (ANLN-Δ3); or both GFP-anillin-Δ3 and HA-Ect2-DHmut (*n* ≥ 42 cells per experiment from *N* = 3 experiments). **g**,**h**, Percentage of cells that entered the confining channels (**g**) and cell entry time (**h**) for HT-1080 cells expressing the constructs in (**f**; *n* = 30 cells per experiment from *N* = 3 experiments). **i**, Frequency of NE rupture in HT-1080 cells expressing GFP-anillin/HA-Ect2 (ANLN/Ect2 (WT)) or GFP-anillin-Δ3 and HA-Ect2-DHmut during migration in confining channels. HT-1080 cells expressing eGFP were used as controls (*n* ≥ 33 cells per experiment from *N* experiments). Values are represented as mean ± s.d. Statistical analysis was performed by a two-tailed unpaired *t*-test (**b**) and one-way ANOVA followed by Tukey’s multiple comparisons test (**d**–**i**) after log transformation (**c**). **j**, Mechanistic model of cell invasion in confinement. Panel **j** created with BioRender.com. Source data

**Extended Data Fig. 4:**
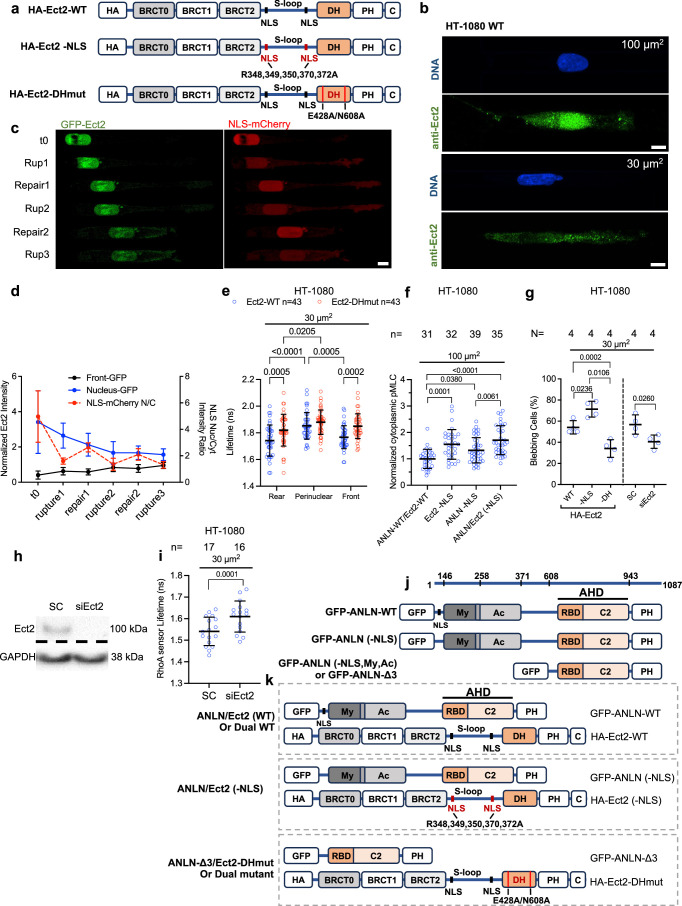

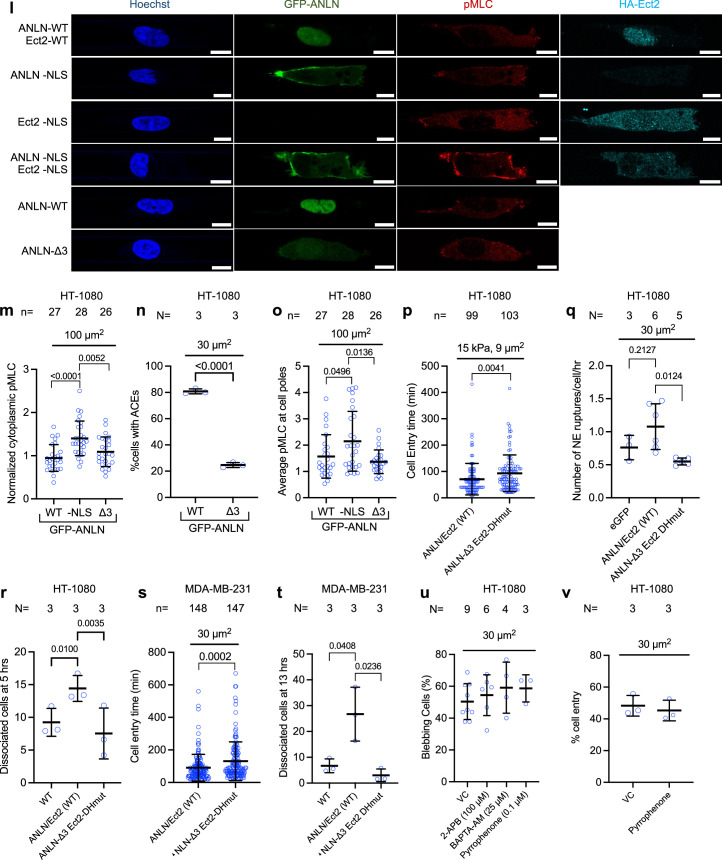
Cytoplasmic localization of anillin and Ect2 promotes RhoA/myosin II-dependent contractility and bleb-based confined cell migration. **(a)** Schematic depicting Ect2 mutant constructs (not in scale). Mutated regions are indicated in red. **(b)** Representative immunofluorescence images of HT-1080 cells inside moderately-confining (100 µm^2^) and confining (30 µm^2^) channels stained with Hoechst 33342 and an anti-Ect2 antibody. **(c)** Representative images of HT-1080 cells showing GFP-Ect2 nuclear exit upon repeated NE ruptures. **(d)** Quantification of front and nuclear GFP-Ect2 signal intensity upon repeated NE rupture and repair cycles, as demonstrated by NLS-mCherry nuclear/cytoplasmic (N/C) signal (n = 10 cells from N = 3 experiments). **(e)** Spatial distribution of RhoA activity in Ect2-WT and Ect2-DHmut cells migrating inside 30 µm^2^ microchannels as measured by FLIM-FRET (n = 43 cells from N = 4 experiments). **(f)** Cytoplasmic pMLC intensity, as assessed from fixed and stained HT-1080 cells expressing either both GFP-anillin and HA-Ect2, or GFP-anillin or HA-Ect2 with a mutated NLS domain, or both GFP-anillin and HA-Ect2 with mutated NLS domains in moderately-confining channels (n cells from N = 3 experiments). **(g)** Percentage of blebbing cells in confining channels for HT-1080 cells ectopically expressing HA-Ect2 (WT), or HA-Ect2 with NLS mutations or HA-Ect2 with DH mutations. In separate experiments, the percentage of blebbing cells in confining channels was calculated for SC or siEct2 HT-1080 cells (n ≥ 25 cells per experiment from N = 4 experiments). **(h)** Western blot showing Ect2 knockdown efficiency via siRNA. GAPDH was used as a loading control. **(i)** Donor fluorescence lifetime of RhoA activity biosensor RhoA2G for HT-1080 cells transiently transfected with either scramble control (SC) or siRNA against Ect2 (siEct2), as measured by FLIM-FRET (n cells from N = 2 experiments). **(j)** Schematic depicting anillin mutant constructs with deletions of different binding domains used in this study (not in scale). **(k)** Schematic showing the combination of anillin and Ect2 mutant constructs in cell lines with dual overexpression used in this study. **(l)** Representative images of pMLC intensity for HT-1080 expressing the indicated constructs and stained with Hoechst, anti-HA and anti-pMLC. **(m,o)** pMLC cytoplasmic intensity (**m**) and its distribution at the cells poles (**o**) inside moderately-confining (100 µm^2^) channels, as assessed from fixed and stained HT-1080 cells expressing either GFP-anillin (WT) or GFP-anillin with a deleted NLS domain, or GFP-anillin with NLS, Myosin, Actin deletion (ANLN-Δ3) (n cells from N = 3 experiments). **(n)** Quantification of the percentage of HT-1080 cells expressing either GFP-ANLN-WT or GFP-ANLN-Δ3 that exhibit ACEs (n ≥ 39 cells per experiment from N = 3 experiments). **(p)** Quantification of cell entry time for HT-1080 expressing GFP-anillin/HA-Ect2 (WT) or the dual mutant during migration in compliant (15 kPa) tightly confining channels (n cells from N = 3 experiments). **(q)** Average number of NE ruptures per cell per hour for HT-1080 cells expressing eGFP (control), GFP-anillin/HA-Ect2 (WT) or the dual mutant ANLN-Δ3/Ect2-DHmut during migration in PDMS-based confining channels (n ≥ 33 cells per experiment from N experiments). **(r)** Number of cells dissociating from spheroids embedded in 3D collagen gels after 5 h for the following cells: HT-1080 WT, GFP-anillin/HA-Ect2 (WT) or the dual mutant cells (n ≥ 10 spheroids per experiment from N = 3 experiments). **(s)** Quantification of cell entry time for MDA-MB-231 expressing GFP-anillin/HA-Ect2 (WT) or GFP-anillin-Δ3/HA-Ect2-DHmut (dual mutant) during migration in PDMS-based confining channels (n cells from N = 3 experiments). **(t)** Number of cells dissociating from spheroids embedded in 3D collagen gels after 13 h for the following MDA-MB-231 cells: WT, GFP-anillin (WT)/HA-Ect2 (WT) or the dual mutant cells (n ≥ 12 spheroids per experiment from N = 3 experiments). **(u)** Percentage of blebbing cells in confining channels for HT-1080 cells treated with vehicle, 2-APB (100 µM), BAPTA AM (25 µM) or pyrrophenone (0.1 µM) (n ≥ 15 cells per experiment from N experiments). **(v)** Percentage of cells that entered confining channels for mCherry-tagged HT-1080 cells expressing GFP-anillin (WT) and HA-Ect2 (WT) under vehicle control (VC) or cPLA_2_ inhibition with pyrrophenone (n ≥ 183 cells per experiment from N = 3 experiment). The HT-1080 and MDA-MB-231 cell lines used in (**p**,**r**,**t**) express mCherry and were sorted for both GFP and mCherry positive cells. All representative images are from single confocal sections. Scale bars: 10 µm. Values represent the mean±S.D. Significance was assessed by two-way ANOVA followed by Sidak’s multiple comparison (**e**), one-way ANOVA followed by Tukey’s multiple comparisons test (g (WT vs -NLS vs -DH),m,q) after log transformation (**f**,**o**), or repeated-measures one-way ANOVA followed by Dunnett’s multiple comparisons (**r**,**t**), or unpaired t-test (g (SC vs siEct2),**i**,**n**) after log transformation (**p**,**s**). Source data

Considering the roles of anillin and the RhoGEF Ect2 in activating RhoA at the contractile ring during cytokinesis^13,16^, we hypothesized that the presence of these proteins in the cytoplasm may contribute to elevated RhoA/myosin II-dependent contractility. First, we examined the subcellular distribution of Ect2 in HT-1080 cells on 2D substrates and in confinement. Like anillin, Ect2 is present in the cytoplasm of cells plated on 2D substrates, as assessed by the immunofluorescence of endogenous or HA-tagged exogenous Ect2 (Fig. 5a and Extended Data Fig. 4a). Similar observations were made for endogenous Ect2 in moderately confining or confining channels (Extended Data Fig. 4b). Unlike anillin, Ect2 or HA-Ect2 only mildly accumulated at the cell edges in a subset of cells on 2D gels and in channels (Fig. 5a and Extended Data Fig. 4b). Although GFP-Ect2 showed more prominent nuclear localization relative to endogenous Ect2 or HA-Ect2, confinement-induced NE rupture was followed by GFP-Ect2 release to the cytoplasm and its moderate accumulation at the plasma membrane (Extended Data Fig. 4c,d and Supplementary Video 11).

To test the Ect2 contribution to RhoA/myosin II-dependent contractility, we expressed different HA-tagged Ect2 mutant constructs in HT-1080 cells. The ectopic expression of Ect2 with mutations in its DH catalytic domain (Extended Data Fig. 4a), which is required for nucleotide exchange on RhoA ^40^, reduced the overall RhoA activity in confining channels relative to wild-type (WT) Ect2, as quantified by increased donor fluorescence lifetimes via FLIM (Fig. 5b), suggesting dominant negative effects on the endogenous RhoA-GTP levels. This mutation also resulted in lower RhoA activity at the cell front and rear (Extended Data Fig. 4e). By contrast, the overexpression of active Ect2 that harbours mutations in its NLS regions^40^ (Extended Data Fig. 4a) increased the cell contractility, as evidenced by immunostaining against the phosphorylated myosin light chain (pMLC) and elevated fluorescence intensity in the cell cytoplasm (Extended Data Fig. 4f). Although the overexpression of Ect2 (-NLS) mutant did not increase pMLC accumulation at leading or trailing cell edges (Fig. 5c), it increased cell blebbing in both moderately confining and confining channels (Fig. 5d and Extended Data Fig. 4g), suggesting that this effect was mediated through an overall increase in actomyosin contractility throughout the cytoplasm. Conversely, impairing the Ect2 activity via mutations in its DH catalytic domain reduced cell blebbing in confinement (Extended Data Fig. 4g). Because the binding of the Ect2-DH mutant to RhoA could potentially inhibit RhoA activation by other RhoGEFs, we also assessed the contribution of Ect2 via siRNA. Consistent with our findings using the Ect2-DH mutant, Ect2 knockdown markedly suppressed confinement-induced RhoA activation and plasma membrane blebbing (Extended Data Fig. 4g–i). Together, these data highlight the key role of Ect2 in regulating RhoA activity and migration phenotype in confinement.

To assess the individual and potentially cooperative roles of anillin and Ect2 in confined migration, we ectopically expressed different mutant constructs of anillin and Ect2 in HT-1080 cells (Extended Data Fig. 4j,k). Deletion of the NLS domain sequestered GFP-anillin in the cytoplasm (Extended Data Fig. 4l) and increased myosin II contractility and cell blebbing in moderately confining channels (Fig. 5d and Extended Data Fig. 4f,m). Interestingly, the ectopic coexpression of anillin and Ect2 NLS mutants led to even more pronounced increases in cell blebbing and myosin II contractility with markedly intense pMLC signals at the front and rear cell edges (Fig. 5c,d and Extended Data Fig. 4f,l), in contrast to the overexpression of the Ect2 NLS mutant alone, presumably due to the increased abundance of cytoplasmic anillin available to locally concentrate RhoA-GTP at cell edges. In particular, the effects of the co-overexpression of the anillin and Ect2 NLS mutants on pMLC emulate the spatial distribution of RhoA/ROCK/myosin II-dependent contractility in confinement (Fig. 1c). Collectively, these data suggest that the cytoplasmic accumulation of anillin and Ect2 activates actomyosin contractility at the cell front and/or rear, and promotes the conversion of cells from a mesenchymal to a blebbing phenotype.

We next sought to elucidate how anillin and Ect2 mediate their effects. We hypothesized that anillin, following confinement-induced cytoplasmic accumulation at cell edges, acts as a subcortical actomyosin-binding scaffold protein that locally concentrates RhoA-GTP^15,41^. Indeed, removing the NLS-, myosin- and actin-binding domains from anillin (anillin-NLS,-My,-Ac and anillin-Δ3 for brevity, respectively; Extended Data Fig. 4j) resulted in a more diffuse distribution of anillin throughout the cytoplasm, revealing the pivotal role of actomyosin in anchoring anillin at the cell poles, which enables ACE formation (Extended Data Fig. 4l,n). Moreover, this triple deletion markedly reduced the percentage of blebbing cells compared with anillin, lacking only its NLS domain in confinement (Fig. 5e). These findings with the triple deletion mutant are also in accordance with the attenuation of cytoplasmic pMLC levels and accumulation at the cell edges observed in moderately confining channels relative to anillin lacking only NLS (Extended Data Fig. 4l,m,o).

Because RhoA/myosin II contractility promoted cell entry into confining microenvironments (Fig. 1g,h) and NE rupture^17^, we examined the roles of anillin and Ect2 in these processes. The ectopic expression of constructs that disrupt either anillin polarization via Δ3 deletions or the Ect2 activity via DH mutations suppressed the percentage of blebbing cells (Fig. 5f) and delayed their entry into stiff, confining channels (Fig. 5g,h) and compliant, tightly confining channels (Extended Data Fig. 4p). The co-overexpression of both mutants resulted in an additive inhibitory effect (Fig. 5f–h), and reduced the percentage of cells displaying NE rupture as well as the frequency of NE rupture events in confinement (Fig. 5i and Extended Data Fig. 4q). To extend the physiological relevance of our findings, we used a 3D spheroid model that mimics the aspects of cell dissociation from a primary tumour and invasion to the surrounding ECM^42,43^. In line with observations in stiff and compliant microchannels, HT-1080 cells expressing the dual anillin and Ect2 mutant exhibited reduced dissociation from 3D spheroids (Extended Data Fig. 4r). To validate our findings in other cancer cell lines, we demonstrate that the MDA-MB-231 cells expressing the dual mutant also displayed a reduced invasive potential, as evidenced by delayed cell entry into stiff, confining channels and markedly decreased cell dissemination from 3D spheroids (Extended Data Fig. 4s,t).

Previous studies demonstrated the role of calcium-dependent signalling in the elevation of contractility in confinement^44,45^. Specifically, the moderate compression of NE along the dorsoventral axis increased the nuclear and endoplasmic reticulum membrane stretching, resulting in the release of calcium from internal membrane stores^44,45^. Intracellular calcium, intracellular stretch-activated calcium channels and the nuclear tension sensor cPLA2 were required for inducing contractility in moderate confinement (*H* = 5 µm)^44,45^. However, the inhibition of this pathway via treatment with BAPTA AM, 2-APB or the cPLA2 inhibitor pyrrophenone had no effect on cell blebbing or cell entry in confined (*H* = 3 µm) channels (Extended Data Fig. 4u,v), suggesting that the effects of confinement-induced RhoA/ROCK/myosin II activation have a more dominant role in promoting confined migration phenotypes relative to the calcium/cPLA2 pathway.

## Anillin and Ect2 drive tumour cell invasion and extravasation in vivo

**Fig. 6:**
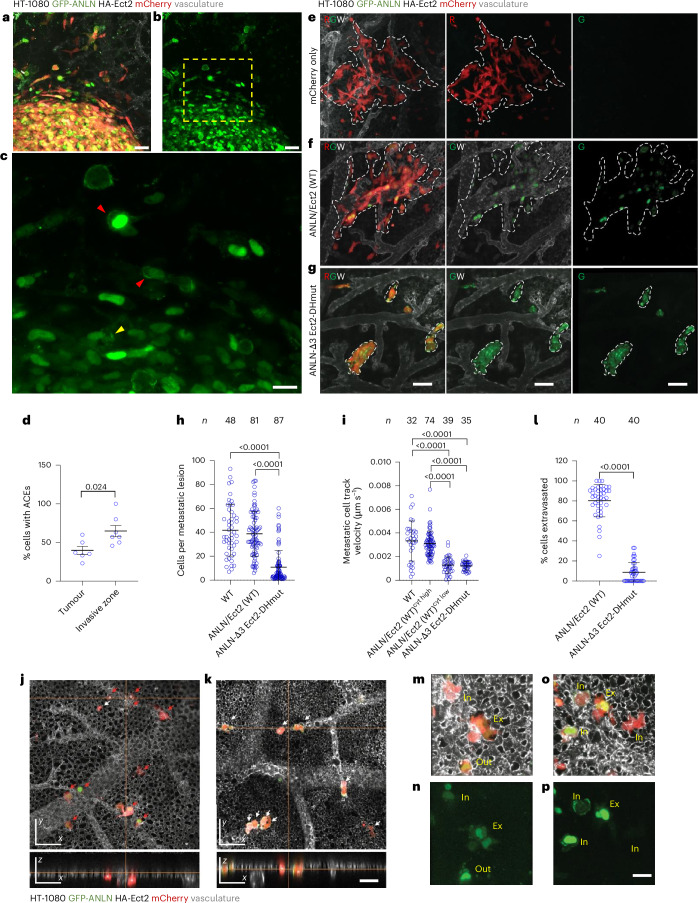
Dual anillin and Ect2 mutation suppresses cell invasion in a chick embryo cancer xenograft model*.* **a**,**b**, Composite (**a**) and GFP image (**b**) of mCherry-tagged HT-1080 cells expressing GFP-anillin (WT)/HA-Ect2 (WT) during invasion into the surrounding tissue from the primary tumour. The dashed yellow box highlights an immediate region surrounding the primary tumour from which cells dissociate. Scale bars, 50 µm. **c**, Magnification of the inset from **b** showing ACEs in cells in the invasion zone (red arrowheads) and the tumour core (yellow arrowheads). Scale bar, 20 µm. **d**, Quantification of ACE frequency in cells in the core versus invasive zone (*n* ≥ 77 cells from six tumours and seven invasive zones from seven animals). **e**–**g**, Images showing metastatic lesions (white dashed lines) formed by mCherry-tagged HT-1080 WT cells (**e**), mCherry-tagged GFP-anillin/HA-Ect2 (WT) cells (**f**) or GFP-anillin-Δ3 and HA-Ect2-DHmut (**g**). Scale bars, 100 µm. **h**, Quantification of the average number of cells in metastatic lesions formed by WT, ANLN/Ect2 (WT) or ANLN-Δ3/Ect2-DHmut HT-1080 cells (*n* lesions from 15 animals). **i**, Quantification of the average track velocity of metastatic HT-1080 WT cells, ANLN/Ect2 (WT) with high or low cytoplasmic anillin, or ANLN-Δ3/Ect2-DHmut cells (*n* cells from four (mCherry only), five (ANLN/Ect2 (WT)) and six (ANLN-Δ3/Ect2-Dhmut) animals). **j**,**k**, Representative images showing HT-1080 ANLN/Ect2 (WT) cells (**j**) or ANLN-Δ3/Ect2-DHmut cells (**k**) extravasating from the CAM vasculature via 3D reconstructions. The red arrows point to extravasated cells; the white arrows indicate cells still inside the vasculature. The top and bottom panels show the *x*–*y* and *x*–*z* views, respectively. Scale bar, 20 µm. **l**, Quantification of the average percentage of extravasated cells (*n* ≥ 20 cells per field of view, two fields per animal, across 20 animals). **m**–**p**, High-magnification images showing HT-1080 ANLN/Ect2 (WT) cells in the process of extravasating out of the CAM vasculature. The bottom panels show GFP-anillin only. In, intravascular; Ex, extravasating; Out, extravasated cells. Exposure in **o** and **p** is increased to show cytoplasmic anillin. Scale bars, 20 µm. Values are represented as mean ± s.d. All images are confocal maximum-intensity *z* projections. Statistical significance was assessed by a two-tailed unpaired *t*-test (**d**), Kruskal–Wallis followed by Dunn’s multiple comparisons (**h**), one-way ANOVA followed by Tukey’s multiple comparisons (**i**) or two-tailed Mann–Whitney test (**l**). Source data

**Extended Data Fig. 5:**
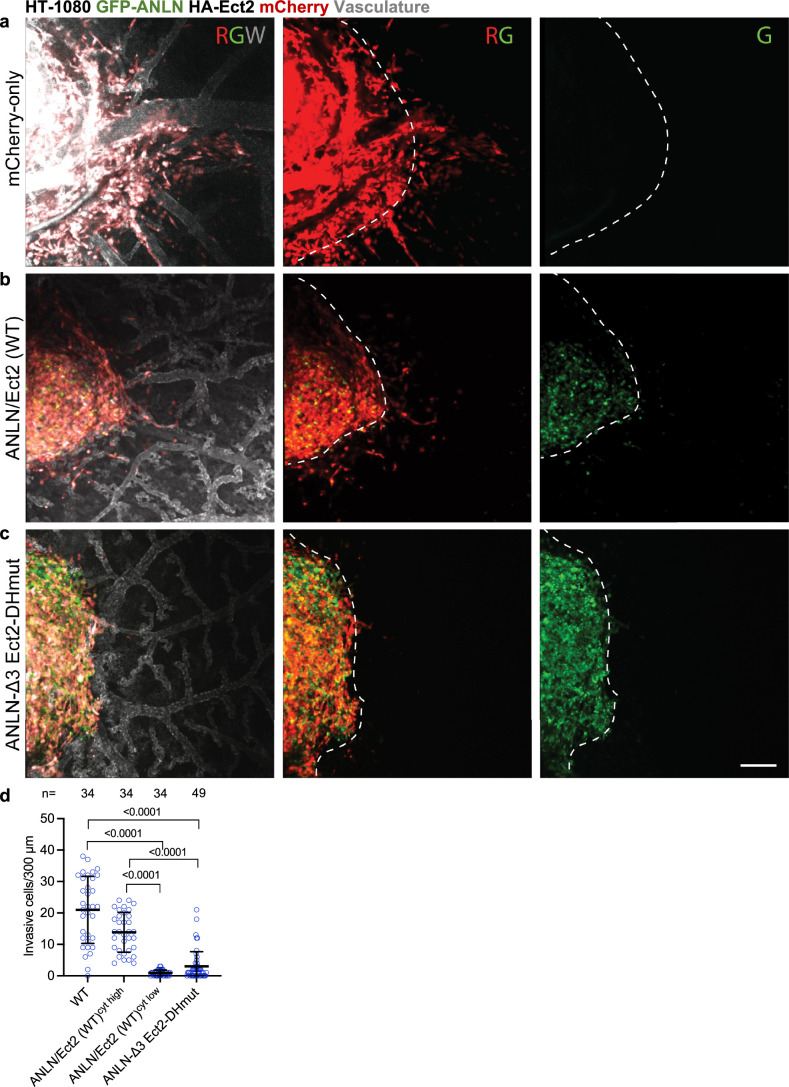
Dual anillin and Ect2 mutation suppresses cell invasion in a chick embryo model*.* **(a-c)** Representative overview (10x) images (confocal maximum z-intensity-projection) showing primary tumors formed by mCherry-tagged HT-1080 WT cells (**a**), mCherry-tagged HT-1080 cells expressing GFP-anillin (WT) and HA-Ect2 (WT) (**b**) or dual mutant (**c**). Dashed white lines denote arbitrary tumor invasion fronts. Scale bars: 100 μm. **(d)** Quantification of the number of invasive cells at the primary tumor invasive zone for HT-1080 WT, GFP-anillin/HA-Ect2 (WT) with high or low cytoplasmic anillin, or ANLN-Δ3/Ect2-DHmut cells (n tumors from N ≥ 10 animals). Values represent the mean±S.D. Statistical significance was assessed by Kruskal-Wallis followed by Dunn’s multiple comparisons test. Source data

To extend our in vitro findings showing that anillin and Ect2 promote cell dissociation from 3D tumour spheroids and confined migration to the in vivo setting, we used the ex ovo chick embryo cancer xenograft model^46–48^. mCherry-labelled HT-1080 cells coexpressing WT GFP-anillin and HA-Ect2 (ANLN/Ect2 (WT)) or anillin and Ect2 dual mutant (ANLN-Δ3/Ect2-DHmut; Extended Data Fig. 4k) were injected between the chick embryo chorioallantoic membrane (CAM) ectoderm and endoderm layers, and their invasion was monitored 5 days post-tumour cell injection using intravital imaging. High-magnification imaging of cells expressing dual WT GFP-anillin and HA-Ect2 (Fig. 6a–c) reveals that, in agreement with the data obtained from mouse in vitro maturation (Fig. 4), a higher proportion of cells that invaded out of the primary tumour displayed anillin accumulation at the cell edges compared with those remaining inside the tumour core (Fig. 6d). This is further substantiated by data showing that cells with high cytoplasmic levels of anillin invade more efficiently into the area surrounding the primary tumours compared with cells with lower cytoplasmic levels of anillin (Extended Data Fig. 5a–d). Moreover, the disruption of both anillin and Ect2 functions profoundly affected the HT-1080 cancer cell invasion, resulting in a minimal number of invasive cells at the primary tumour front (Extended Data Fig. 5b–d).

To assess the contributions of anillin and Ect2 in the formation of metastatic colonies, HT-1080 cells expressing dual WT (ANLN/Ect2 (WT)) or dual mutants of anillin and Ect2 (ANLN-Δ3/Ect2-DHmut; Extended Data Fig. 4k) were injected intravenously into the CAM vasculature of chick embryos^46^ and allowed to form metastatic colonies for 4 days. Although the WT-anillin- and Ect2-overexpressing HT-1080 cells formed rapidly growing invasive metastatic lesions, comparable with control HT-1080 cells expressing mCherry (Fig. 6e,f,h), the anillin and Ect2 dual mutants formed compact non-invasive lesions that contained a smaller number of cells (Fig. 6e–h and Supplementary Video 12). Intriguingly, metastatic cells overexpressing ANLN/Ect2 (WT) but displaying low cytoplasmic anillin levels migrated markedly slower than those with high cytoplasmic anillin (Fig. 6i). Mutations of both anillin and Ect2 also resulted in similarly decreased cancer cell track velocities (Fig. 6i). Together, these findings suggest that the minimal invasive activity observed in cells overexpressing ANLN/Ect2 (WT) but displaying low cytoplasmic anillin levels (Extended Data Fig. 5a–d) results from impaired cell migration, reaching levels similar to those detected in cells expressing mutations in both anillin and Ect2 (Fig. 6i).

Cancer cell extravasation requires the directional extension of invadopodia that are necessary for forced vascular wall breaching^3^. We, thus, tested if the disruption of both anillin and Ect2 functions is detrimental to successful cancer cell extravasation. Indeed, the anillin and Ect2 dual mutant cells extravasated poorly relative to WT cells (Fig. 6k,l). High-magnification visualization of dual WT cells also revealed an increase in the cytoplasmic signal of anillin, which was observed inside the vasculature during extravasation and persisted even after the extravasation process was complete (Fig. 6m–p). These data suggest that spatial confinement imposed on cells during extravasation in vivo, as well as physical cues present in the vasculature, such as fluid shear stress, regulate the redistribution of anillin and Ect2, resulting in RhoA activation, which is critical for efficient cell invasion and extravasation.

## Conclusions

**Extended Data Fig. 6:**
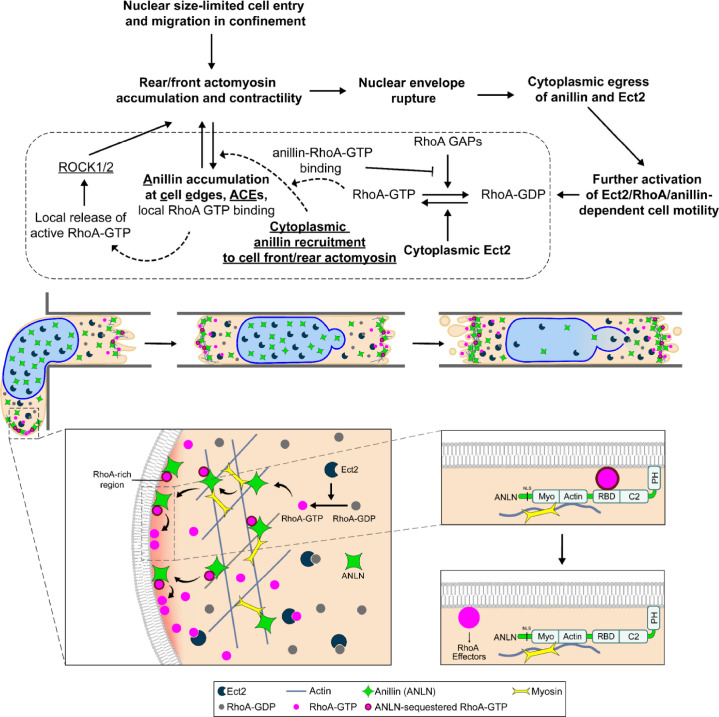
Proposed mechanism for cytoplasmic anillin and Ect2 regulation of actomyosin contractility and cell invasiveness. Cell entry into confinement induces actomyosin accumulation at the rear and front cell edges, which leads to concomitant local recruitment of cytoplasmic anillin through binding to actomyosin. While the cytoplasmic pool of RhoA GEF Ect2 mediates GTP loading on RhoA, the RBD domain of actomyosin-bound anillin captures and locally concentrates RhoA-GTP in a low-affinity complex^49^. Upon dissociation from the anillin scaffold, RhoA-GTP can interact with downstream effectors such as ROCK1/2, thereby locally activating actomyosin contractility to promote cell invasion. Mechanical forces acting on nuclei during confined migration, including actomyosin contractility-induced nuclear influx and nuclear blebbing^17^, lead to NE rupture and de-regulated mixing of nuclear and cytoplasmic contents. The NE rupture-induced cytoplasmic egress of anillin and Ect2 further exacerbates RhoA-myosin II contractility. Bold letters indicate parts of the pathways that were elucidated in this study.

On the basis of our findings, we propose the following mechanistic model (Fig. 5j and Extended Data Fig. 6) for anillin/Ect2-mediated RhoA activation at the leading and trailing edges of cells migrating in confinement. First, consistent with our live-cell imaging data, physical cues induced by the nuclear entry to confinement trigger a concomitant inflow of F-actin and the recruitment of anillin to plasma-membrane-proximate actomyosin zones, resulting in the formation of sharply delineated micrometre-sized pools with locally highly concentrated anillin, ACEs, at the leading and rear cell edges. Although accumulating within ACEs by binding to actomyosin via its N-terminal domains, anillin can simultaneously bind, via its C-terminal RhoA-binding domain, RhoA-GTP previously generated by cytoplasmic Ect2. Importantly, previous structural and biochemical evidence indicates that when bound to anillin, RhoA-GTP is inaccessible to its upstream regulators, such as Ect2 or RhoA GTPase-activating proteins (GAPs), as well as to their downstream effectors, including ROCK kinases^25^. At the same time, the relatively low affinity of anillin and RhoA-GTP binding (~7 µM)^49^ predicts that RhoA-GTP probably dissociates readily from ACEs in its free form capable of activating ROCK and other effectors, or becoming inactivated via GAPs. In light of these previous studies, our results suggest that the anillin at cell edges, whose local levels reach or exceed its nuclear levels, could act as a high-concentration/low-affinity scaffolding ‘sponge’ that locally breaks the symmetry of RhoA activators/inhibitors by shielding RhoA-GTP from the abundant GAPs, and enabling the released RhoA-GTP to mediate actomyosin activation. The actomyosin contractility, activated through the ACE-dependent process at the cell edges, propels cell entry to confinement, eventually leading to NE rupture induced by mechanical forces acting on the confined nucleus^17,50^. The resulting release of nuclear anillin and Ect2 into the cytoplasm further augments ACE formation, amplifying the roles of anillin and Ect2 in cell migration through positive feedback. As such, anillin and Ect2 work together to hyperactivate cellular contractility and facilitate cell entry into confining channels.

In summary, our studies reveal a direct and active role of anillin as a dynamically regulated, multifaceted scaffold that tunes, in coordination with Ect2, RhoA-dependent actomyosin contractility to the posterior and anterior edges of migrating cells in confinement. In line with the notion that Rho/ROCK and actomyosin contractility are drivers of metastasis^51^, we found that the disruption of anillin and Ect2 function suppresses cancer cell invasion at the tumour fronts and extravasation.

## Methods

Mouse studies were approved by the Institutional Animal Care and Use Committee of The University of Texas, MD Anderson Cancer Center, which is accredited by the Association for Assessment and Accreditation of Laboratory Animal Care (IACUC protocol 00001002). All procedures involving the chick embryo model were approved by the University of Alberta Institutional Animal Care and Use Committee.

### Cell culture and pharmacological inhibitors

Human HT-1080 fibrosarcoma cells, MDA-MB-231 breast cancer cells, A431 epidermoid carcinoma and human foreskin fibroblasts (HFF-1) cells were acquired from American Type Culture Collection. HOS cells were obtained from the NIH AIDS Research and Reference Reagent Program (Division of AIDS, NIAID, NIH). HT-1080, MDA-MB-231, HOS, A431 and HFF-1 cells were cultured in Dulbecco’s modified Eagle’s medium (DMEM) containing 4.5 g l^–1^ glucose, l-glutamine and sodium pyruvate (Gibco) and supplemented with 10% heat-inactivated foetal bovine serum (Gibco, 16140071) and 1% penicillin/streptomycin (10,000 U ml^–1^; Gibco, 15140122).

BRC-196 breast cancer cells (kindly provided by Dr. Seagel at McGill University) were cultured in DMEM/F-12 containing 3.151 g l^–1^ of glucose, 15 mM of HEPES, l-glutamine and sodium pyruvate supplemented with 10% heat-inactivated foetal bovine serum (Gibco, 16140071), 10 nM of β-estradiol (Sigma, E8875), 0.4 µg ml^–1^ of hydrocortisone (MilliporeSigma, H0135), 5 ng ml^–1^ of heregulin-β1 (STEMCELL Technologies, 78071) and 4 µg ml^–1^ of insulin (Sigma, I9278). Cells were grown in an incubator maintained at 37 °C and 5% CO_2_, and subcultured every 2–4 days.

In select experiments, cells were treated with the following pharmacological agents and the corresponding vehicle controls. Reagents were obtained from Sigma-Aldrich unless otherwise noted: Y27632 (Y0503, 10 µM), hydroxyurea (H8627, 8 mM), blebbistatin (B0560, 50 µM), GM6001 (364206, 20 µM), 2-APB (Tocris Bioscience, 1224, 100 µM), BAPTA AM (Invitrogen, B6769, 25 µM), pyrrophenone (Cayman Chemical, 13294, 0.1 µM), importazole (SML0341, 20 or 40 µM), doxycycline (D3072, 10 nM), paraformaldehyde solution (Electron Microscopy Sciences, 1517-S), Triton X-100 (T9284), Tween-20 (P7949), Nonidet P-40 substitute (VWR, M158), Everyblot blocking buffer (BioRad, 12010020), TBS (Quality Biological, 351-086-101) and bovine serum albumin (A7030).

### Photolithography and device fabrication

PDMS microfluidic devices, consisting of an array of parallel channels with a fixed channel length of 200 µm and different heights and widths were fabricated as described previously^52–54^. On the basis of their cross-sectional areas, channels were classified as moderately confining (*W* × *H* = 10 × 10 µm^2^), confining (*W* × *H* = 10 × 3 µm^2^) or tightly confining (*W* × *H* = 3 × 3 µm^2^). For cell migration experiments, channels were coated with 20 µg ml^–1^ of rat tail collagen I (Gibco, A1048301).

### Microfluidic device seeding and live-cell imaging

Cell seeding was performed as described previously^17^. To create a chemotactic gradient, the bottom three wells of each device were filled with serum-free DMEM (1% penicillin/streptomycin), whereas the top well was filled with serum-containing DMEM (10% FBS and 1% penicillin/streptomycin).

### Cell phenotype analysis

Cells were allowed to migrate in PDMS-based channels for 4–5 h at which timepoint, the cells were fixed and stained with Hoechst 33342 and phalloidin (as detailed below) and observed using a Nikon AXR confocal with ×40 water objective or an inverted Nikon Eclipse Ti microscope using a ×40 air objective. Migration phenotype was manually tabulated using the criteria described in refs. ^11,17^.

To calculate the percentage of cell entry, we counted the total number of cells within a distance of 50 µm from the channel entrances and quantified the fraction of these cells that fully entered the microchannels. Cell entry time was defined as the duration between cell protrusions first extending into the interior of the microchannels and full cell entry.

### Effects of nuclear import and RhoA activity on cytoplasmic anillin level

HT-1080 cells were seeded into 96-well glass-bottomed plates (Cellvis, P96-1.5H-N) at 4,000 cells per well. After 24 h, cells were treated with either importazole for nuclear import inhibition or doxycycline for inducible overexpression of GFP-RhoA (Q63L). Following 24 h of treatment, cells were washed and fixed with warm 4% paraformaldehyde in phosphate-buffered saline, stained for endogenous anillin and imaged with an ImageXpress Micro Confocal high-content microscope (Molecular Devices) similar to the protocol described above.

### Live-actin imaging

Cells harvested from flasks were resuspended in a medium containing 1:1,000 SPY650-FastAct (Cytoskeleton; CY-SC505, 1,000× stock in dimethyl sulfoxide), seeded into microfluidic devices, and kept for 3.5 h in an incubator before imaging.

### Fluorescence imaging and quantification

All fluorescence data, except for those in Fig. 2d, Extended Data Fig. 2a and Fig. 5a, were acquired on a Nikon A1 or AXR confocal microscopes (Nikon) using a ×63 oil objective with a numerical aperture of 1.4 or ×40 water objective with a numerical aperture of 1.15. Also, 640-nm, 567-nm, 488-nm and 405-nm lasers were used for imaging. Fluorescence intensity was quantified using ImageJ (v.2.16.0/1.54p). For anillin, the front and rear plasma membranes and the nucleus were selected as depicted in Extended Data Fig. 2d. Fluorescence intensity was measured in selected regions and normalized to the total cell fluorescence intensity. In 3D collagen and viscoelastic alginate gels, the cell pole with the highest fluorescence signal was quantified and identified as the cell periphery.

For pMLC quantification, fluorescence intensities for the whole cell, nucleus, and front and rear plasma membranes were quantified. Cytoplasmic pMLC intensity is the mean fluorescence of the cytoplasmic area excluding the nucleus (Extended Data Fig. 2d). Mean fluorescence of the cell front and rear is normalized to the mean cytoplasmic intensity excluding the nucleus and the front and rear membranes.

### ACE quantification

Cell *z* scans were taken at 0.5-µm intervals, and the *z* plane with the strongest membrane anillin signal was chosen. GFP-anillin intensity was analysed along a 4-pixel-wide linescan running between the membrane region displaying anillin signal and the cytoplasm. Cells on 2D, 3D and moderately confining channels are rendered positive for ACE if they have ≥5 µm of membrane region with anillin intensity at least twice that of the surrounding cytoplasmic region. Similarly, in confining channels, ACE-positive cells are considered those with at least twice the anillin intensity signal at the cell poles relative to the surrounding cytoplasmic region.

### NE rupture imaging and quantification

Cells expressing NLS-mCherry were imaged on a Nikon A1 confocal microscope using a Plan Apo ×20 air objective with a numerical aperture of 0.75 and a resolution of 1,024 × 512 pixel^2^. A central *z* plane of cells inside a confining channel or *z* stacks at a 0.5-µm interval were acquired. For the quantification of NE ruptures, the reduction in nuclear NLS-mCherry signal with the corresponding increase in its cytoplasmic intensity is considered a rupture event. Conversely, the recovery of the nuclear signal accompanied by a reduction in the cytoplasmic signal is marked as an NE repair event. Cells were analysed from when their nuclei reached the channel entrances until the cell protrusions reached the channel exit. Cells whose nuclei were obstructed by particles or cellular debris during confined migration were excluded.

The videos of migrating cells in confinement were manually inspected to identify the the *x* and *y* positions of the centres of nuclei in the first frame corresponding to full nuclear entry into microchannels. The nuclei images were first segmented using a custom program developed in MATLAB_R2023b^55–57^. The area (*A*), perimeter (*P*), and the long-axis and short-axis lengths of the segmented nuclei were then computed using the image processing toolbox in MATLAB. The aspect ratio was calculated as the ratio of the long-axis length and the short-axis length, and the circularity is 4π*A*/*P*^2^.

Manual linescan analyses with ImageJ were used to identify video frames indicating NE rupture events as signalled by the abrupt increase in the cytoplasmic signal and decrease in the nuclear NLS-mCherry signal. In parallel, a 10-pixel-wide linescan with ImageJ in the GFP-anillin channel was used to identify video frames corresponding to the formation of front or rear ACEs. Time stamps of all changes in the nuclear/cytoplasmic NLS-mCherry and ACE detection in the GFP-anillin channel were recorded in Excel (v.16.97.2) sheets and used for the calculation of the frequency of NE ruptures and timing of the front and rear ACEs with respect to NE rupture. Similarly, linescans of the nuclear and diffuse perinuclear cytoplasmic GFP-anillin with ImageJ were used to detect the timing of nuclear GFP-anillin exit.

### FLIM of RhoA FRET sensors

Confocal FLIM of live cells that were stably expressing the RhoA2G sensor was performed as described previously^17^ using a ZEISS LSM 780 microscope and a PicoQuant system consisting of the PicoHarp 300 time-correlated single-photon-counting module, two hybrid PMA-04 detectors and a Sepia II laser control module.

### FLIM image processing, segmentation and quantification

The FLIM data were processed as described previously^17^ using SymPhoTime 64 (PicoQuant) software. Pseudocolour heat-map images showing the fluorescent lifetimes within predefined ranges were prepared with a customized ShowFluorLifeData MATLAB R2023a script^55^.

### HEMICA device preparation and seeding

An array of parallel channels with a fixed channel length of 200 µm and different heights and widths were designed via soft photolithography, as described previously^54,58^. HEMICA microchannels with elastic moduli of 8 kPa and 21 kPa were fabricated, and coated with collagen type I, as previously described^31^.

### Alginate gel preparation, fabrication and mechanical testing

High-molecular-weight I1G alginate (~260 kDa) was purchased from KIMICA and was irradiated by a cobalt-60 source to produce low-molecular-weight alginate (~27 kDa). RGD-coupled alginate was prepared by coupling the peptide GGGGRGDSP (Peptide 2.0) using carbodiimide chemistry. The alginate was then purified by dialysis (3,500 molecular-weight cut-off) against deionized water containing sodium chloride for 3 days, treated with activated charcoal, sterile filtered, lyophilized and reconstituted in DMEM (1% penicillin/streptomycin, no FBS) following previously reported methods^34^. Calcium sulfate (CaSO_4_) was mixed with alginate as a source for the release of crosslinking calcium ions. The mixture was transferred to a glass plate coated with Sigmacote, covered and allowed to gel for 45 min. Gel discs, which were 15 mm in diameter and 2 mm thick, were equilibrated in DMEM (1% penicillin/streptomycin, no FBS) for 24 h before mechanical testing. The elastic modulus and stress relaxation properties of alginate hydrogels were measured by compression tests of the gel discs using an MTS Criterion Series 40 Tensile Tester. The gel discs were compressed to 15% strain at a deformation rate of 2 mm min^–1^ and a 100-Hz data acquisition rate. For the relaxation process, the compression strain was kept at 15%, as the load was recorded over time. The elastic modulus was derived from the slope of the linear region of the stress–strain curve (~5–10% of strain). Stress relaxation properties were quantified by relaxation half-time (*t*_1/2_), which is the time for the initial stress to be relaxed to half its value during stress relaxation test.

### Tumour implantation and intravital multiphoton microscopy

Athymic nu/nu female mice were obtained from the Department of Experimental Radiation Oncology, M.D. Anderson Cancer Center. Dorsal skin-fold chambers were mounted on 8-to-12-week-old female athymic nu/nu mice as described previously^35^. In brief, the skin-fold chamber was mounted on a skin flap on the back to cover the deep dermis after surgically removing the opposite side of the skin. One day post-surgery, pelleted HT-1080 cells (2.5–5 × 10^5^ cells in 2–4 µl) stably expressing NLS-mCherry and GFP-anillin were injected into the dermis with a 30-G needle. Three tumours per chamber were implanted and monitored for up to 11 days. Intravital microscopy was performed on a LaVision TrimScope II scanner with three titanium–sapphire lasers (Chameleon-XR, Coherent) and two optical parametric oscillators compact systems (APE/Coherent; tunable excitation wavelengths range between 800 nm and 1,300 nm) on days 2–11 to monitor the tumour growth and subcellular distribution of NLS-mCherry and GFP-anillin. Next, mice were anaesthetized with isoflurane (1–3% in oxygen), placed on a temperature-controlled stage (37 °C) and the chamber was mounted on a holder. Blood vessels were visualized by a retro-orbital injection of 70-kDa dextran (Invitrogen/Thermo Fisher) labelled with Alexa Fluor 750 (1 mg per mouse). Imaging was performed using an Olympus XLPLN25XWMP2 ×25 water objective (numerical aperture, 1.05; working distance, 2 mm). Sequential 3D stacks were acquired with three excitation wavelengths (880 nm, 1,090 nm and 1,280 nm) in two consecutive scans. Emission was detected using the following band-pass filters: third-harmonic generation (1,280 nm; ET450/60 nm), mCherry (1,090 nm; ET595/40 nm), SHG (1,090 nm; ET525/50 nm), Alexa Fluor 750 (1,280 nm; ET810/90 nm) and GFP (880 nm; ET525/50 nm; Chroma Technology). The 3D volumes were acquired for up to a 250-µm penetration depth at a step size of 5 µm. Time-lapse recording with a frame interval of 20 min was performed for a maximum duration of 5 h. The 3D image stacks were reconstructed as the maximum-intensity *z* projection, stitched and analysed using NIH ImageJ.

### In vivo image analysis

To quantify the NE rupture events in vivo, the mean grey values of the the N/C ratio for the NLS-mCherry channel were analysed from 2–3 *z*-projection image slices of the time-lapse sequences and plotted as the signal intensity profiles over time. NLS positivity in the cytoplasm was determined by an average N/C ratio of ≤2.5. The redistribution of anillin to the plasma membrane was quantified as the ratio between the membrane and cytoplasmic (M/C) mean grey values for GFP-anillin. Regions of interest were defined by manual segmentation along the inside of the nucleus and cytoplasm or along the cell edge. Anillin positivity at the membrane was defined as having at least 5 µm of membrane region with the anillin intensity averaging over time at least twice the intensity of the surrounding cytoplasmic region.

To correlate the percentage of cells with cytoplasmic anillin with the geometry of the local invasion environment, the constriction width was quantified using combined 3D SHG, fluorescent dextran and cell-based fluorescence in the orthogonal direction from the invasion path. Only cells bordered by a detectable SHG signal above the background were analysed. We associated these data with the number of cells displaying ACEs in each invasion zone, with ACE quantification performed as described in the relevant section. This analysis was validated through an independent, blinded review.

### Ex ovo chick embryo cancer xenograft model

Fertilized White Leghorn chicken eggs, acquired from the University of Alberta Poultry Research Centre, were maintained in a humidified incubator at 38 °C. After 4 days of incubation, the embryos were removed from their shells and maintained under shell-less conditions in a covered dish at 38 °C and 60% humidity, as previously described^46–48^.

For the primary tumour or primary tumour invasive front imaging, day-10 chicken embryos were injected with 1 × 10^5^ mCherry-labelled HT-1080 cells or mCherry-labelled HT-1080 cells expressing either WT GFP-anillin and HA-Ect2 (ANLN/Ect2 (WT)) or anillin and Ect2 dual mutant (ANLN-Δ3/Ect2-DHmut) in phosphate-buffered saline directly in between the CAM ectoderm and endoderm layers. Sterilized, circular coverslips (22 mm in diameter) were positioned on top of the tumour 1 day post-tumour cell inoculation, and image acquisition was performed 4–5 days later^46–48^.

For metastatic colony imaging, day-10 chicken embryos were injected intravenously with 2.5 × 10^4^ mCherry-labelled HT-1080 cells or mCherry-labelled HT-1080 cells expressing either ANLN/Ect2 (WT) or ANLN-Δ3/Ect2-DHmut. Sterilized, circular coverslips (22 mm in diameter) were positioned on the CAM surface above metastatic colonies 1 day post-tumour cell injection. Metastatic colonies were allowed to develop for four more days, and single metastatic colonies were selected for visualization and analysis^46–48^.

In cancer cell extravasation experiments, 5 × 10^4^ mCherry-labelled HT-1080 cells expressing either ANLN/Ect2 (WT) or ANLN-Δ3/Ect2-DHmut were injected into the CAM vasculature. Cancer cell extravasation was analysed 8 h post-injection, as described in ref. ^59^.

### Image acquisition and analysis

The real-time imaging of cancer cell invasion was performed by acquiring a four-dimensional image series of single cancer cells within the CAM tissue, as previously described^46–48^, using a Nikon A1r upright microscope fitted with a temperature-regulated enclosure and a range of Nikon microscope objectives (×10, ×25 (water immersion) and ×63 (oil)). For the time-lapse analysis, time 0 was defined as the time of the first image capture. Here 20–50 individual cells were tracked (for each cell line used in the experiments) using a built-in object-tracking module in Volocity. The track velocity was calculated as the average speed of the track. The track displacement rate (productivity) was calculated using a built-in Volocity module as the total track displacement (straight-line distance from the first track position to the last) divided by the track time. Cancer cells were identified as anillin cytoplasmic high if the ratio of average anillin signal intensity (GFP) within the nucleus to the average anillin signal intensity within the cytoplasm was ≤3; the cells were identified as cytoplasmic low if the GFP-anillin nuclear/cytoplasmic ratio was ≥5. To quantify the cancer cell number per colony, ×25 *z*-stack images were acquired (2–5-µm step), and the cancer cells were manually counted using Nikon Elements software (v.5.21.00). For the quantification of invasive cancer cells at the primary tumour periphery, individual (×25) *z*-stack images were analysed using the Nikon Elements software. For anillin-GFP signal intensity quantifications, the primary tumour images (main tumour mass and invasive zone) were acquired at ×60 magnification. All the experimental data were plotted and analysed for statistical significance using the Prism analysis module.

Also, ×63 confocal images of the main tumour mass and invasive zones were obtained to quantify the anillin fluorescence intensity ratios. ImageJ was used to quantify the anillin or mCherry fluorescence intensity within the nucleus or cytoplasm within single optical slices (1 µm). For time-lapse tracking analysis, image drift was corrected using the ImageJ Stack_Reg plugin (Biomedical Imaging Group; http://bigwww.epfl.ch/thevenaz/stackreg/).

ACE quantification was performed using ImageJ. For cells inside the primary tumour masses, a 256 × 256-pixel^2^ region of interest was placed in the middle of the tumour mass. All the cells with at least half their nuclei inside the region of interest were included in the analysis. All cells that were found in the invasive zones were analysed. ACE quantification was performed as described in the relevant section. Cells that had no mCherry signal or whose NE was not distinguishable were excluded from our analysis.

Supplementary Methods contain additional information on the following experimental procedures: cloning, lentivirus preparation, transduction, and transfection; actin staining, immunofluorescence imaging and quantification; cell-cycle synchronization; flow cytometry analysis; western blotting; coimmunoprecipitation assays; and collagen- and alginate-gel-related assays.

### Statistics and reproducibility

Data represent the mean ± standard deviation (s.d.) or median with 95% confidence interval from *N* independent experiments. All the experiments were performed in triplicate (three biological replicates), unless otherwise specified. The D’Agostino–Pearson or Shapiro–Wilk omnibus normality test was used to determine whether data are normally or log-normally distributed. Datasets with Gaussian distributions were compared using a two-tailed Student’s *t*-test, a one-way analysis of variance (ANOVA) test followed by a Tukey’s test for multiple comparisons or a two-way ANOVA test followed by a Sidak’s test for multiple comparisons. A Wilcoxon matched-pairs signed rank test was used to determine the statistical significance. Log-normal data were transformed using the *Y* = log[*Y*] formula before comparison. For non-Gaussian distributions, a non-parametric two-tailed Mann–Whitney test was used for comparing two conditions, whereas more than two groups were compared by a Kruskal–Wallis test followed by Dunn’s multiple comparisons. Analysis was performed using GraphPad Prism (v. 6, 7, 8, 9 or 10) software.

### Reporting summary

Further information on research design is available in the Nature Portfolio Reporting Summary linked to this article.

## Online content

Any methods, additional references, Nature Portfolio reporting summaries, source data, extended data, supplementary information, acknowledgements, peer review information; details of author contributions and competing interests; and statements of data and code availability are available at 10.1038/s41563-025-02269-9.

## Supplementary information

Supplementary Information Supplementary Figs. 1–3, Methods and references.

Reporting Summary

Supplementary Video 1 Colocalization of GFP-anillin and actin at the cell poles during entry into confinement. Representative time-lapse recordings of four GFP-anillin-expressing HT-1080 cells labelled with SPY650-FastAct, showing the accumulation and colocalization of anillin and actin, first at the cell rear and then at both poles during cell entry and migration in the confining channels. Exposure was uniformly increased to highlight ACEs before cell entry. Scale bars, 10 µm.

Supplementary Video 2 Colocalization of GFP-anillin and actin at the cell poles during entry into confinement. Representative time-lapse recordings of four GFP-anillin-expressing HT-1080 cells labelled with SPY650-FastAct, showing the accumulation and colocalization of anillin and actin, first at the cell rear and then at both poles during cell entry and migration in the confining channels. Exposure was uniformly increased to highlight ACEs before cell entry. Scale bars, 10 µm.

Supplementary Video 3 Colocalization of GFP-anillin and actin at the cell poles during entry into confinement. Representative time-lapse recordings of four GFP-anillin-expressing HT-1080 cells labelled with SPY650-FastAct, showing the accumulation and colocalization of anillin and actin, first at the cell rear and then at both poles during cell entry and migration in the confining channels. Exposure was uniformly increased to highlight ACEs before cell entry. Scale bars, 10 µm.

Supplementary Video 4 Colocalization of GFP-anillin and actin at the cell poles during entry into confinement. Representative time-lapse recordings of four GFP-anillin-expressing HT-1080 cells labelled with SPY650-FastAct, showing the accumulation and colocalization of anillin and actin, first at the cell rear and then at both poles during cell entry and migration in the confining channels. Exposure was uniformly increased to highlight ACEs before cell entry. Scale bars, 10 µm.

Supplementary Video 5 Cells displaying ACEs before their entry in confinement and enrichment after NE rupture. Representative time-lapse recording of an extended G1/S-phase HT-1080 cell expressing GFP-anillin (WT) and NLS-mCherry entering and migrating inside a confining channel. Note the ACEs before cell entry and their enrichment following NE rupture. Exposure was uniformly increased to highlight ACEs before cell entry. Scale bars, 10 µm.

Supplementary Video 6 Cells in confinement experiencing multiple NE ruptures. Representative time-lapse recording of an extended G1/S-phase HT-1080 cell expressing GFP-anillin (WT) and NLS-mCherry inside a confining channel. Exposure was uniformly increased to highlight ACEs. Scale bar, 10 µm.

Supplementary Video 7 Initiation of NLS-mCherry leakage correlated with further anillin enrichment to the cell cytoplasm and membrane in vivo. Representative time-lapse recording of a confined HT-1080 cell localized near the tumour edge, displaying front and rear ACEs that are further enriched on NE rupture, as shown by GFP-anillin (top; purple heat-map lookup table (LUT)) and NLS-mCherry (bottom; RGB rainbow LUT) exit from the nucleus and localization to the cytosol. Purple arrowheads, anillin on the plasma membrane and transient decrease in NLS intensity in the nucleus and increase in the cytoplasm. Scale bar, 20 µm.

Supplementary Video 8 Anillin accumulation on the plasma membrane at the cell front and/or rear without signs of clear NE rupture in vivo. Representative time-lapse recordings of confined HT-1080 cells localized near the tumour edge, displaying front and rear ACEs even without NE ruptures, as shown by GFP-anillin (top; purple heat-map LUT) and NLS-mCherry (bottom; RGB rainbow LUT). Purple arrowheads show anillin on the plasma membrane. Scale bar, 20 µm.

Supplementary Video 9 Anillin accumulation on the plasma membrane at the cell front and/or rear without signs of clear NE rupture in vivo. Representative time-lapse recordings of confined HT-1080 cells localized near the tumour edge, displaying front and rear ACEs even without NE ruptures, as shown by GFP-anillin (top; purple heat-map LUT) and NLS-mCherry (bottom; RGB rainbow LUT). Purple arrowheads show anillin on the plasma membrane. Scale bar, 20 µm.

Supplementary Video 10 Anillin accumulation on the plasma membrane at the cell front and/or rear without signs of clear NE rupture in vivo. Representative time-lapse recordings of confined HT-1080 cells localized near the tumour edge, displaying front and rear ACEs even without NE ruptures, as shown by GFP-anillin (top; purple heat-map LUT) and NLS-mCherry (bottom; RGB rainbow LUT). Purple arrowheads show anillin on the plasma membrane. Scale bar, 20 µm.

Supplementary Video 11 Nuclear GFP-Ect2 exits to the cytoplasm in confinement following NE ruptures. Representative time-lapse recording of an HT-1080 cell expressing GFP-Ect2 and NLS-mCherry entering and migrating inside confining channels, showing the accumulation of GFP-Ect2 in the cytoplasm as NE ruptures. Scale bars, 10 µm.

Supplementary Video 12 Impact of anillin on cell invasion in vivo. mCherry-tagged HT-1080 cells expressing GFP-anillin-Δ3 and HA-Ect2-DHmut dual mutants formed more compact, less invasive lesions than GFP-anillin (WT)/HA-Ect2(WT) controls. The top panels show mCherry and GFP channels; the bottom panels show the GFP channel only. 20 min per frame; 7.6-h total duration; ×10 magnification. Scale bar, 50 µm.

## Source data

Source Data Fig. 1 Source data for all panels in Fig. 1.

Source Data Fig. 2 Source data for all panels in Fig. 2.

Source Data Fig. 3 Source data for all panels in Fig. 3.

Source Data Fig. 4 Source data for all panels in Fig. 4.

Source Data Fig. 5 Source data for all panels in Fig. 5.

Source Data Fig. 6 Source data for all panels in Fig. 6.

Source Data Extended Data Fig. 1 Source data for all panels in Extended Data Fig. 1.

Source Data Extended Data Fig. 2 Source data for all panels in Extended Data Fig. 2.

Source Data Extended Data Fig. 3 Source data for all panels in Extended Data Fig. 3.

Source Data Extended Data Fig. 4 Source data for all panels in Extended Data Fig. 4.

Source Data Extended Data Fig. 5 Source data for all panels in Extended Data Fig. 5.

## Data Availability

The data that support the findings of this study are available within the Article and its Supplementary Information and are available from the corresponding authors upon request. Source data are provided with this paper.

## Code availability

The codes used for the nuclear morphology analysis and FLIM pseudocolour display are available via Zenodo at 10.5281/zenodo.15178932 (ref. ^55^), with the instructions and license of use available via GitHub at https://github.com/DeepBioVision/Cell-in-Channel-anal.

## References

[1] Paul, C. D., Mistriotis, P. & Konstantopoulos, K. Cancer cell motility: lessons from migration in confined spaces. *Nat. Rev. Cancer* **17**, 131–140 (2017). 27909339 10.1038/nrc.2016.123 PMC5364498

[2] Weigelin, B., Bakker, G.-J. & Friedl, P. Intravital third harmonic generation microscopy of collective melanoma cell invasion. *IntraVital* **1**, 32–43 (2012). 29607252 10.4161/intv.21223 PMC5858865

[3] Hashizume, H. et al. Openings between defective endothelial cells explain tumor vessel leakiness. *Am. J. Pathol.* **156**, 1363–1380 (2000). 10751361 10.1016/S0002-9440(10)65006-7 PMC1876882

[4] van Helvert, S., Storm, C. & Friedl, P. Mechanoreciprocity in cell migration. *Nat. Cell Biol.* **20**, 8–20 (2018). 29269951 10.1038/s41556-017-0012-0 PMC5943039

[5] Fritz, G., Just, I. & Kaina, B. Rho GTPases are over-expressed in human tumors. *Int. J. Cancer* **81**, 682–687 (1999). 10328216 10.1002/(sici)1097-0215(19990531)81:5<682::aid-ijc2>3.0.co;2-b

[6] Petrie, R. J. & Yamada, K. M. Multiple mechanisms of 3D migration: the origins of plasticity. *Curr. Opin. Cell Biol.* **42**, 7–12 (2016). 27082869 10.1016/j.ceb.2016.03.025 PMC5064813

[7] Charras, G. & Paluch, E. Blebs lead the way: how to migrate without lamellipodia. *Nat. Rev. Mol. Cell Biol.* **9**, 730–736 (2008). 18628785 10.1038/nrm2453

[8] Sahai, E. & Marshall, C. J. Differing modes of tumour cell invasion have distinct requirements for Rho/ROCK signalling and extracellular proteolysis. *Nat. Cell Biol.* **5**, 711–719 (2003). 12844144 10.1038/ncb1019

[9] Liu, Y.-J. et al. Confinement and low adhesion induce fast amoeboid migration of slow mesenchymal cells. *Cell* **160**, 659–672 (2015). 25679760 10.1016/j.cell.2015.01.007

[10] Petrie, R. J., Koo, H. & Yamada, K. M. Generation of compartmentalized pressure by a nuclear piston governs cell motility in a 3D matrix. *Science* **345**, 1062–1065 (2014). 25170155 10.1126/science.1256965 PMC5248932

[11] Wisniewski, E. O. et al. Dorsoventral polarity directs cell responses to migration track geometries. *Sci. Adv.* **6**, eaba6505 (2020). 32789173 10.1126/sciadv.aba6505 PMC7399493

[12] Hetmanski, J. H. R. et al. Membrane tension orchestrates rear retraction in matrix-directed cell migration. *Dev. Cell* **51**, 460–475.e410 (2019). 31607653 10.1016/j.devcel.2019.09.006 PMC6863396

[13] Basant, A. & Glotzer, M. Spatiotemporal regulation of RhoA during cytokinesis. *Curr. Biol.* **28**, R570–R580 (2018). 29738735 10.1016/j.cub.2018.03.045 PMC6508076

[14] Tatsumoto, T., Xie, X., Blumenthal, R., Okamoto, I. & Miki, T. Human ECT2 Is an exchange factor for Rho GTPases, phosphorylated in G2/M phases, and involved in cytokinesis. *J. Cell Biol.* **147**, 921–928 (1999). 10579713 10.1083/jcb.147.5.921 PMC2169345

[15] Frenette, P. et al. An anillin-Ect2 complex stabilizes central spindle microtubules at the cortex during cytokinesis. *PLoS ONE* **7**, e34888 (2012). 22514687 10.1371/journal.pone.0034888 PMC3325936

[16] Piekny, A. J. & Glotzer, M. Anillin is a scaffold protein that links RhoA, actin, and myosin during cytokinesis. *Curr. Biol.* **18**, 30–36 (2008). 18158243 10.1016/j.cub.2007.11.068

[17] Mistriotis, P. et al. Confinement hinders motility by inducing RhoA-mediated nuclear influx, volume expansion, and blebbing. *J. Cell Biol.* **218**, 4093–4111 (2019). 31690619 10.1083/jcb.201902057 PMC6891075

[18] Tuan, N. M. & Lee, C. H. Role of anillin in tumour: from a prognostic biomarker to a novel target. *Cancers* **12**, 1600 (2020). 32560530 10.3390/cancers12061600 PMC7353083

[19] Cook, D. R. et al. Aberrant expression and subcellular localization of ECT2 drives colorectal cancer progression and growth. *Cancer Res.* **82**, 90–104 (2022). 34737214 10.1158/0008-5472.CAN-20-4218 PMC9056178

[20] Wang, D., Naydenov, N. G., Dozmorov, M. G., Koblinski, J. E. & Ivanov, A. I. Anillin regulates breast cancer cell migration, growth, and metastasis by non-canonical mechanisms involving control of cell stemness and differentiation. *Breast Cancer Res.* **22**, 3 (2020). 31910867 10.1186/s13058-019-1241-x PMC6947866

[21] Fritz, R. D. et al. A versatile toolkit to produce sensitive FRET biosensors to visualize signaling in time and space. *Sci. Signal.* **6**, rs12 (2013). 23882122 10.1126/scisignal.2004135

[22] Denais, C. M. et al. Nuclear envelope rupture and repair during cancer cell migration. *Science* **352**, 353–358 (2016). 27013428 10.1126/science.aad7297 PMC4833568

[23] Raab, M. et al. ESCRT III repairs nuclear envelope ruptures during cell migration to limit DNA damage and cell death. *Science* **352**, 359–362 (2016). 27013426 10.1126/science.aad7611

[24] Chen, A., Akhshi, T. K., Lavoie, B. D. & Wilde, A. Importin β2 mediates the spatio-temporal regulation of anillin through a noncanonical nuclear localization signal. *J. Biol. Chem.* **290**, 13500–13509 (2015). 25829492 10.1074/jbc.M115.649160 PMC4505596

[25] Budnar, S. et al. Anillin promotes cell contractility by cyclic resetting of RhoA residence kinetics. *Dev. Cell* **49**, 894–906 e812 (2019). 31105010 10.1016/j.devcel.2019.04.031

[26] Beaudet, D., Pham, N., Skaik, N. & Piekny, A. Importin binding mediates the intramolecular regulation of anillin during cytokinesis. *Mol. Biol. Cell* **31**, 1124–1139 (2020). 32238082 10.1091/mbc.E20-01-0006 PMC7353161

[27] Soderholm, J. F. et al. Importazole, a small molecule inhibitor of the transport receptor importin-beta. *ACS Chem. Biol.* **6**, 700–708 (2011). 21469738 10.1021/cb2000296 PMC3137676

[28] Mistriotis, P., Wisniewski, E. O., Si, B. R., Kalab, P. & Konstantopoulos, K. Coordinated in confined migration: crosstalk between the nucleus and ion channel-mediated mechanosensation. *Trends Cell Biol.* **34**, 809–825 (2024). 38290913 10.1016/j.tcb.2024.01.001 PMC11284253

[29] Cantwell, H. & Dey, G. Nuclear size and shape control. *Semin. Cell Dev. Biol.* **130**, 90–97 (2022). 34776332 10.1016/j.semcdb.2021.10.013

[30] Apraiz, A., Mitxelena, J. & Zubiaga, A. Studying cell cycle-regulated gene expression by two complementary cell synchronization protocols. *J. Vis. Exp.*10.3791/55745 (2017). 10.3791/55745 PMC5608252 28654080

[31] Afthinos, A. et al. Migration and 3D traction force measurements inside compliant microchannels. *Nano Lett.* **22**, 7318–7327 (2022). 36112517 10.1021/acs.nanolett.2c01261 PMC9872269

[32] Cox, T. R. & Erler, J. T. Remodeling and homeostasis of the extracellular matrix: implications for fibrotic diseases and cancer. *Dis. Model Mech.* **4**, 165–178 (2011). 21324931 10.1242/dmm.004077 PMC3046088

[33] Wolf, K. et al. Collagen-based cell migration models in vitro and in vivo. *Semin. Cell Dev. Biol.* **20**, 931–941 (2009). 19682592 10.1016/j.semcdb.2009.08.005 PMC4021709

[34] Chaudhuri, O. et al. Hydrogels with tunable stress relaxation regulate stem cell fate and activity. *Nat. Mater.* **15**, 326–334 (2016). 26618884 10.1038/nmat4489 PMC4767627

[35] Haeger, A. et al. Collective cancer invasion forms an integrin-dependent radioresistant niche. *J. Exp. Med.* **217**, e20181184 (2020). 31658985 10.1084/jem.20181184 PMC7037234

[36] Kammoun, M. et al. Development of a novel multiphysical approach for the characterization of mechanical properties of musculotendinous tissues. *Sci. Rep.* **9**, 7733 (2019). 31118478 10.1038/s41598-019-44053-1 PMC6531478

[37] Wang, N. et al. Cell prestress. I. Stiffness and prestress are closely associated in adherent contractile cells. *Am. J. Physiol. Cell Physiol.* **282**, C606–C616 (2002). 11832346 10.1152/ajpcell.00269.2001

[38] Van Rijssel, J. et al. The Rho-GEF Trio regulates a novel pro-inflammatory pathway through the transcription factor Ets2. *Biol. Open* **2**, 569–579 (2013). 23789107 10.1242/bio.20134382 PMC3683159

[39] Kazanietz, M. G., Barrio-Real, L., Casado-Medrano, V., Baker, M. J. & Lopez-Haber, C. The P-Rex1/Rac signaling pathway as a point of convergence for HER/ErbB receptor and GPCR responses. *Small GTPases* **9**, 297–303 (2018). 27588611 10.1080/21541248.2016.1221273 PMC5997144

[40] Huff, L. P. et al. The role of Ect2 nuclear RhoGEF activity in ovarian cancer cell transformation. *Genes Cancer* **4**, 460–475 (2013). 24386507 10.1177/1947601913514851 PMC3877668

[41] Piekny, A. J. & Maddox, A. S. The myriad roles of anillin during cytokinesis. *Semin. Cell Dev. Biol.* **21**, 881–891 (2010). 20732437 10.1016/j.semcdb.2010.08.002

[42] Bera, K. et al. Extracellular fluid viscosity enhances cell migration and cancer dissemination. *Nature* **611**, 365–373 (2022). 36323783 10.1038/s41586-022-05394-6 PMC9646524

[43] Zhang, Y. et al. Polarized NHE1 and SWELL1 regulate migration direction, efficiency and metastasis. *Nat. Commun.* **13**, 6128 (2022). 36253369 10.1038/s41467-022-33683-1 PMC9576788

[44] Lomakin, A. J. et al. The nucleus acts as a ruler tailoring cell responses to spatial constraints. *Science* **370**, eaba2894 (2020). 33060332 10.1126/science.aba2894 PMC8059074

[45] Venturini, V. et al. The nucleus measures shape changes for cellular proprioception to control dynamic cell behavior. *Science* **370**, eaba2644 (2020). 33060331 10.1126/science.aba2644

[46] Yankaskas, C. L. et al. The fluid shear stress sensor TRPM7 regulates tumor cell intravasation. *Sci. Adv.* **7**, eabh3457 (2021). 34244134 10.1126/sciadv.abh3457 PMC8270498

[47] Leong, H. S. et al. Invadopodia are required for cancer cell extravasation and are a therapeutic target for metastasis. *Cell Rep.* **8**, 1558–1570 (2014). 25176655 10.1016/j.celrep.2014.07.050

[48] Stoletov, K. et al. Quantitative in vivo whole genome motility screen reveals novel therapeutic targets to block cancer metastasis. *Nat. Commun.* **9**, 2343 (2018). 29904055 10.1038/s41467-018-04743-2 PMC6002534

[49] Sun, L. et al. Mechanistic insights into the anchorage of the contractile ring by anillin and Mid1. *Dev. Cell* **33**, 413–426 (2015). 25959226 10.1016/j.devcel.2015.03.003 PMC4449299

[50] Xia, Y. et al. Nuclear rupture at sites of high curvature compromises retention of DNA repair factors. *J. Cell Biol.* **217**, 3796–3808 (2018). 30171044 10.1083/jcb.201711161 PMC6219729

[51] Rodriguez-Hernandez, I., Cantelli, G., Bruce, F. & Sanz-Moreno, V. Rho, ROCK and actomyosin contractility in metastasis as drug targets. *F1000Res.*10.12688/f1000research.7909.1 (2016). 10.12688/f1000research.7909.1 PMC4856114 27158478

[52] Wong, B. S. et al. A direct podocalyxin-dynamin-2 interaction regulates cytoskeletal dynamics to promote migration and metastasis in pancreatic cancer cells. *Cancer Res.* **79**, 2878–2891 (2019). 30975647 10.1158/0008-5472.CAN-18-3369 PMC6548656

[53] Yankaskas, C. L. et al. A microfluidic assay for the quantification of the metastatic propensity of breast cancer specimens. *Nat. Biomed. Eng.* **3**, 452–465 (2019). 31061459 10.1038/s41551-019-0400-9 PMC6563615

[54] Zhao, R. et al. Cell sensing and decision-making in confinement: the role of TRPM7 in a tug of war between hydraulic pressure and cross-sectional area. *Sci. Adv.* **5**, eaaw7243 (2019). 31355337 10.1126/sciadv.aaw7243 PMC6656542

[55] Wu, P. H. DeepBioVision: cell in channel analysis. *Zenodo*10.5281/zenodo.15178932 (2025).

[56] Wu, P. H. et al. Single-cell morphology encodes metastatic potential. *Sci. Adv.* **6**, eaaw6938 (2020). 32010778 10.1126/sciadv.aaw6938 PMC6976289

[57] Wu, P. H. et al. Evolution of cellular morpho-phenotypes in cancer metastasis. *Sci. Rep.* **5**, 18437 (2015). 26675084 10.1038/srep18437 PMC4682070

[58] Balzer, E. M. et al. Physical confinement alters tumor cell adhesion and migration phenotypes. *FASEB J.* **26**, 4045–4056 (2012). 22707566 10.1096/fj.12-211441 PMC3448771

[59] Willetts, L., Bond, D., Stoletov, K. & Lewis, J. D. Quantitative analysis of human cancer cell extravasation using intravital imaging. *Methods Mol. Biol.* **1458**, 27–37 (2016). 27581012 10.1007/978-1-4939-3801-8_3

