# Supplementary material for: Cytoplasmic anillin and Ect2 promote RhoA/myosin II-dependent confined migration and invasion
Source: Nat Mater. 2025 Jun 26;24(9):1476–88. doi: 10.1038/s41563-025-02269-9 (PMC12404997; doi:10.1038/s41563-025-02269-9)
Supplement: Supplementary file 1 — Supplementary Figs. 1–3, Methods and references. [file 41563_2025_2269_MOESM1_ESM.pdf]

nature materials

Supplementary information https://doi.org/10.1038/s41563-025-02269-9

Cytoplasmic anillin and Ect2 promote RhoA/myosin II-dependent confined migration and invasion

In the format provided by the authors and unedited

Table of Contents for Supplementary Materials

The PDF file includes:

Supplementary Methods

References

Supplementary Figures S1 to S3

Other Supplementary Materials for this manuscript include the following:

Supplementary Videos 1 to 12

Supplementary Methods

Cloning, lentivirus preparation, transduction, and transfection- To generate shRNA lentiviral vectors, the target sequences were subcloned into pLVTHM (Addgene, Cambridge, MA, plasmid # 12247) using MluI and ClaI as restriction sites or pLKO.1 (Addgene, Cambridge, MA, plasmid #8453) using AgeI and EcoRI as restriction sites. The target sequences are:

Scramble Control: sh1 (GCACTACCAGAGCTAACTCAGATAGTACT),
human MYH9 (ACGGAGATGGAGGACCTTATG),
human MYH10 (GGATCGCTACTATTCAGGA).

WT GFP-Anillin and GFP-Anillin_^{68}AAA^{70} plasmids were kindly provided by the Wilde Lab[1] (University of Toronto, Toronto, Ontario, Canada). GFP-Ect2 was generously given by the Yap Lab[2] (The University of Queensland, St. Lucia, Brisbane, Queensland, Australia). Anillin mutant constructs (GFP-anillin lacking NLS, NLS and myosin, NLS, actin, and myosin, NLS and AHD) were kindly provided by the Glotzer Lab[3] (University of Chicago, Chicago, Illinois). pFugW-HA-Ect2 wild type and mutants (NLS- and DH- mutant Ect2) were generously given by the Cox Lab[4] (University of North Carolina at Chapel Hill, Chapel Hill, NC). The following plasmids were purchased from Addgene: pLenti.PGK.LifeAct-GFP.W (plasmid #51010), pLenti.PGK.H2B-mCherry (plasmid # 51007), pQC NLS mCherry IX (plasmid #37354), pEGFP-Anillin (plasmid #68027), tetO-FUW-eGFP-RHOA-Q63L (plasmid #73081), RhoA2G FRET biosensor (plasmid #40176, #40179), psPAX2 (plasmid #12260), and pMD2.G (plasmid #12259), VSV.G (plasmid #14888), pLV-EF1α-IRES-puro (plasmid #85132), pLV-EF1α-IRES-Hygro (plasmid #85134).

Transient transfections (**Fig. 5e**, and **Extended Data Fig. 4g,i**) as well as lentivirus production and infection were performed as described previously[5, 6].

For siRNA knockdown, scramble (sc-37007) and Ect2 (sc-35259) siRNA were purchased from Santa Cruz Biotechnology. Cells were transiently transfected with siRNA using the Lipofectamine RNAiMax Kit (Invitrogen, 13778075) according to the manufacturers protocol.

Actin staining, immunofluorescence imaging and quantification- For actin staining related to cell blebbing analysis, cells were fixed with 4% paraformaldehyde (PFA) (Affymetrix, Inc.), permeabilized in 0.1% Triton X-100 (Sigma), and blocked in 1% bovine serum albumin (Sigma). Cells were stained with rhodamine or Alexa Fluor 488 (AF488) phalloidin (1:100, Invitrogen #R415 or A12379) and Hoechst (1:4000 - 1:2500, Invitrogen #H1399).

For IF in **Fig. 2d** and **Extended Data Fig. 2a**, cells were grown in 96-well glass-bottom plates (Cellvis, P96-1.5H-N) and fixed with 4% PFA (Electron Microscopy Sciences 157-14-S) in PBS, pH 7.4, for 10-15 min at room temperature (RT). Samples were permeabilized and DNA-stained

by treatment with 1% Triton X-100 (Sigma #P7949), 0.05% Tween-20 (Sigma), 20 µg/ml Hoechst 33342 for 3-5 min, incubated for 30-60 min at RT with blocking buffer (10% normal donkey serum (Millipore-Sigma S30-100ml), 0.05% Tween20, 0.05% NaN_3 (Sigma S2002) in PBS, pH 7.4), all at RT, before overnight incubation (at 4ºC) with primary antibodies (rabbit anti-anillin (Sigma-Aldrich, HPA005680, 1:200), mouse anti-RhoA (Santa Cruz Biotechnology, sc-418, 1:100) diluted in blocking buffer. Similarly, for endogenous Ect2 or stably expressed HA-Ect2 staining on 2D (**Fig. 5a**), cells were seeded on 96-well glass-bottom plates (Cellvis, P96-1.5H-N), and fixed and processed as above, except that EveryBlot reagent (BioRad #12010020) was used for blocking (100%, 10-15 min) and primary antibodies (rabbit anti-Ect2, EMD Millipore. 07-1364; 1:100, or rabbit anti-HA, Cell Signaling Technology #3724; 1:100) were incubated overnight (4ºC) in 0.05% Tween20, 0.05% NaN_3 in TBS, pH7.4 (TTBS).

After incubation with secondary antibodies and phalloidin (Alexa Fluor 488 donkey anti-rabbit (Invitrogen A32790n, 1:1000), Alexa Fluor 555 Donkey anti-mouse (Invitrogen A32773, 1:1000), Alexa Fluor 647 Phalloidin (Invitrogen A22297, 1:400) and washes with TTBS, all at RT, the wells were supplemented with 50 µl TTBS and plates imaged with ImageExpress Micro Confocal high content microscope (Molecular Devices), with 20x air objective in spinning disc confocal mode with 60 µm pinhole. A 3x3 grid of images per each well contained ~400-9000 individual cell images/well. MetaXpress software was used to analyze the local background-corrected mean cytoplasmic to nuclear ratios (C/N) in raw, unaltered images, using the translocation-enhanced module (Molecular Devices) with parameters adjusted to individual immunfluorescence channels. The results were filtered to exclude non-physiological values (mean intensity >0, N/C ratios <100) and processed via Microsoft Excel PivotChart.

ImageJ was used to uniformly subtract image specific out-of-cell fluorescence for each channel before assembling representative IF images (**Fig. 2d, Extended Data Fig. 2a**) with Adobe Photoshop 2023. Linescan analyses (10 µm-wide) in **Fig. 2a,b** were prepared with ImageJ and colored with GraphPad Prism 10.

For endogenous Ect2 staining in PDMS devices (**Extended Data Fig. 4b**), cells were seeded and allowed to migrate for 5 h before being washed and fixed with warm 8-10% PFA in PBS for 20 min. Next, cells were washed 3x with D-PBS, permeabilized with 0.5% Triton-X100, blocked with 1 M glycine + 0.1% NP-40 in PBS for 20 min followed by 15 min blocking with BioRad Everyblot blocking buffer. Samples were then incubated overnight at 4°C with rabbit anti-Ect2 antibody (EMD Millipore, 07-1364, 1:100) diluted in antibody dilution buffer (0.05% BSA, 0.05% Tween-20,

0.1% Triton-X100, 0.05% NaN_3 in TBS). 24 h later, samples were washed 3x with D-PBS + 0.1% NP-40 before incubation with Alexa Fluor 488 or 568 goat anti-rabbit antibody and Hoechst 33342 presented in antibody dilution buffer.

For immunofluorescence in the remaining figures, cells were fixed with 4% PFA (Thermo Fisher), permeabilized in 0.1% or 0.2% Triton X-100, and blocked in 5% BSA (Sigma, A7030)/0.05% Tween-20/0.05% NaN_3 or 5% Bovine Serum Albumin (BSA) (Sigma)/2% Normal Goat Serum (Cell Signaling Technology 5425S)/0.2% Triton X-100. Samples were incubated overnight at 4°C with the following primary antibodies: anillin (Sigma-Aldrich, HPA005680, 1:100)), HA (Cell Signaling, 2367, 1:600), or phospho-myosin light chain 2 (Cell Signaling, 3671S, 1:50) antibody. Afterwards, samples were washed 3x with PBS.

The following secondary antibodies were used: Goat Anti-Rabbit IgG H&L, Alexa Fluor 488 (ThermoFisher, A11034, 1:200), Goat Anti-Rabbit IgG H&L, Alexa Fluor 568 (ThermoFisher, A11011, 1:200), Goat Anti-Mouse IgG H&L, Alexa Fluor 488 (ThermoFisher, A11001, 1:200), and Goat Anti-Mouse IgG H&L, Alexa Fluor 568, (ThermoFisher, A21043, 1:200). All antibodies were prepared in blocking buffer.

Cell cycle synchronization- For early G1/S-phase synchronization, cells were serum-starved in 0.25% FBS for 48 h, then released with full media (DMEM containing 10% FBS and 1% P/S) 30 min before the experiment[7]. For extended G1/S-phase synchronization, cells were serum-starved in 0.25% FBS for 48 h, released with full media for 5 h, then treated with 8 mM hydroxyurea for 3 h before the experiment[7]; cells were maintained in 8 mM hydroxyurea throughout the microfluidic experiment.

Flow cytometry analysis- 800,000 GFP-anillin-expressing HT-1080 cells were synchronized as described above, kept in DMEM medium containing either 2.5% FBS (early G1/S) or 2.5% FBS and 8 mM hydroxyurea (extended G1/S) for 5 h, trypsinized, centrifuged, and resuspended in 500 µl D-PBS. Samples were briefly kept on ice, then fixed with 4.5 ml of ice-cold 70% ethanol. To stain with propidium iodide (PI), samples were centrifuged at 300 g, 4°C for 5 min, washed with 5 ml ice-cold D-PBS, centrifuged again and incubated for 30 min at 37°C in $2x10^6$ cells/ml of staining solution: 20 µg/ml PI (Sigma P4864), 200 µg/ml RNase A (Invitrogen, 8003089), 0.1% Triton X-100 (Sigma, T9284) in PBS. Samples were analyzed on a BD FACSCanto Flow Cytometer at the Johns Hopkins Integrated Imaging Center.

Analysis was done in FlowJo v10. Three sequential gatings (FSC-A vs SSC-A, FSC-A vs FSC-H, PI-A vs PI-W) were performed to identify the PI-positive population. Cell cycles were identified using FlowJo univariate Watson (Pragmatic) model algorithm.

Western blotting- To perform Western Blots in different cell lines (**Fig. 2e**), sub-confluent cell cultures were harvested by trypsinization. Following trypsin deactivation with FBS, cell pellets were washed 2X in D-PBS (Gibco, 14190-144) before resuspension in 4% SDS, 120 mM Tris/HCl, pH 6.8, immediately followed by heating at 100ºC for 5 min. The lysates were sonicated (Branson Sonifier 250, power 4, 5s), protein concentration measured with Micro BCA Protein Assay Kit (Thermo Pierce, 23235) and samples stored at -80ºC or used immediately for western blotting. Before electrophoresis, samples were reconstituted at 1 µg/µl in reducing 2x SDS Laemmli Sample buffer (Thermo, J61337.AC) and heated at 100ºC for 3 min. Samples (10 µg lysate/well) were separated on 4-20% SDS PAGE Criterion gels and blotted to PVDF membrane with TransBlot Turbo (BioRad). After 60 min blocking with 5% Blotting Grade Blocker (BioRad, 1706404) prepared in TTBS, blots were incubated with primary antibodies diluted in TTBS overnight at 4ºC (rabbit anti-anillin (Sigma-Aldrich, HPA005680, 1:1000 combined with mouse anti-RhoA (Santa Cruz Biotechnology, sc-418, 1:200) and ECL (Thermo, SuperSignal West Femto, 34095) images developed after incubating with rabbit antibodies (HRP-linked anti rabbit, Cell Signaling Technology, 7074, 1:5000, 20 min). After inhibiting the HRP with 30% H_2O_2 (Millipore Sigma, H1009, 30 min at RT), the blots were developed with mouse antibodies (HRP-linked anti mouse, Cell Signaling Technology 7076, 1:5000, 20 min). The blots were stained with 25% Isopropanol, 10% Acetic Acid, 0.05% Coomassie R 250, washed with water, dried, imaged and uniformly background-substracted with Image J to verify equal total protein abundance between samples.

Western Blots for **Extended Data Fig. 4h** were performed as previously described[8] using NuPage 3-8% or 4-12% gels and the following antibodies: Primary antibodies: mouse anti-Ect2 (Santa Cruz, sc-514750, 1:100), rabbit GAPDH (Cell Signaling Technology, 2118, 1:1000) was used as loading control. Secondary antibodies: Anti-mouse IgG, HRP-linked Antibody (Cell Signaling Technology, 7076S, 1:2000), anti-rabbit IgG, HRP-linked antibody (Cell Signaling Technology, 7074S; 1:2000).

Anillin co-immunoprecipitation with active RhoA. HT-1080 cell line stably expressing GFP-RhoA Q639L from tetO-FUW-eGFP-RHOA-Q63L were grown in two 15-cm culture dishes until 70-80% confluency. The plates were washed twice with DPBS and the growth medium (DMEM, 10% FBS) replaced with DMEM, 0.1% FBS. Interphase cells were obtained by 60-72 h of serum

starvation and subsequent release into DMEM, 10% FBS for 4 h before harvesting by trypsinization.

A modification of the nuclear/cytoplasmic fraction preparation[9] was applied to obtain nuclear fraction-free cytoplasmic extracts, using digitonin permeabilization instead of Igepal C-630 (NP-40) to permeabilize plasma membranes. The cells were washed by centrifugation (400 g, 2 min, RT) with D-PBS, resuspended in 15 ml 10 mM sodium phosphate, pH 7.4, proteinase inhibitor cocktail, EDTA-free (PIC, Roche 59813300), kept on ice for 10 min to induce hypo-osmotic swelling, pelleted at 400 g, 4 min, RT and supplemented with 2 mM $MgCl_2$, 5 mM GTP (pH 7.4). 40 mg/ml stock of digitonin (Sigma, D141) in DMSO was added in 50 µg/ml increments (final concentration) to reach >60-80% release of nuclei, as judged by bright field microscopy, typically requiring 200 µg/ml. A 30 µl aliquot of the permeabilized cell suspension was lysed with 1% NP-40 (final concentration, total cell extract, TCEx) and the rest loaded in 200-300 µl aliquots on top of 1ml sucrose cushions (10 mM Tris pH 7.4, 150 mM NaCl, 24% sucrose, PIC) in 1.7 ml Eppendorf tubes and centrifuged at 1500 g, 8 min, 4ºC. The pellet (enriched in nuclei but containing cytoplasmic contamination) was rinsed with D-PBS, PIC, lysed in 1% NP-40, D-PBS, pH 7.4, PIC, resulting in nuclear extract, NEx). The supernatant (cytoplasmic extract thoroughly depleted of nuclear contamination, CEx) was clarified of all remaining nuclei by three spins at 1500 g, 2 min, 4ºC each. Next, the clarified CEx was combined with pelleted and DPBS- washed 50 µl GFP-trap (Proteintech/Chromotech; gtma-20) beads or 50 µl binding control magnetic agarose (Proteintech/Chromotech; bmab, non-specific binding control). After incubation on a rotator at 4ºC for 1.5 h, the beads were collected on a magnetic stand, supernatant removed, the beads washed 4X with 800 µl ice-cold TTBS, 0.05% NP40, transferred to new Eppendorf tubes and washed 2X. After removing the washing buffer, the beads were collected in 15 µl 2x SDS Laemmli sample buffer and heated for 5 min at 100ºC. The protein concentration in TCEx, NEx and CEx was measured with BioRad Protein Assay (BioRad, 50000006). Aliquots of the equal protein amounts of the TCEx, NEx and CEx (3 µg each), along with the total of control- or GFP-trap bead elutions were separated on 4-20% SDS PAGE Criterion gels (BioRad), blotted to PVDF and developed with rabbit anti-anillin antibodies as described above. A portion of the blots corresponding to ~5-25kDa proteins was subsequently developed with rabbit anti-histone H3 antibodies (Cell Signalling Techology, 4499, 1:2000) to verify the absence of nuclear proteins in the cytoplasmic fractions used for the IP. After blocking HRP with H_2O_2 (see above), the blot was re-developed with mouse anti-GFP antibody (Roche, 11814460001, 1:1000) to verify successful pulldown of GFP-RhoA(Q63L).

Collagen gel preparation and imaging- In select experiments, rat tail collagen I (Corning) was diluted in 0.1% acetic acid to form a 7.1 mg/mL stock collagen solution. To form collagen gels with embedded cells, appropriate volumes of collagen, 1x M199 Medium (Thermo Fisher), 10x M199 Medium, and cells were mixed on ice to create a solution with final concentrations of 2.5 mg/mL collagen I, 1x M199, and 2 x 10^5 cells/mL. 200 mM NaOH was added incrementally while mixing on ice until a pH of 7.5 was obtained. In other experiments, rat tail collagen I (Corning #354236) was diluted to 2.5 mg/ml using 10x low-glucose DMEM (Sigma #D2429) and 1x complete DMEM media, and neutralized with 10 M NaOH on ice.

60 µL of this solution were pipetted into select wells of a glass-bottomed 96-well plate and incubated for 20 min at 37°C to allow gel formation, then 200 µL of DMEM (1% penicillin/streptomycin, 10% FBS) with or without GM6001 (EMD Millipore, 20 µM) were added to each well. To form collagen gels with cells on the surface, the above gel formulation procedure was repeated omitting cells, and 40 µL of this solution were pipetted into wells of a glass 96-well plate. After the 20 min gelation, 200 µL DMEM (1% penicillin/streptomycin, 10% FBS) containing 5 x 10^4 cells/mL with or without GM6001 were added to the gel surface. After two days, media for all conditions was replaced, likewise with or without GM6001. On the third day, cells were imaged using a Zeiss LSM 800 confocal microscope using a 20X air objective and a resolution of 1024X1024 pixels. 567-nm and 488-nm lasers were used for imaging. Images were processed using Zen Blue software and ImageJ.

Cell dissociation from collagen gel-embedded spheroid- To form spheroids, 2,000 HT-1080 or 3,000 MDA-MB-231 cells were resuspended in 150 µl of ice-cold 2% v/v Matrigel (Corning #356230) in complete media (DMEM containing 10% FBS), added into round bottom ultra-low attachment 96-well plate (Corning, 7007), centrifuged for 5 min at 300 g, and then incubated at 37°C and 5% CO_2 for 2 days. Collagen gel was formed by mixing 1 ml of rat tail collagen I (Corning, 354236) with 125 µl of 10x low-glucose DMEM (Sigma, D2429) and neutralized with ≥3.3 µl of 10 M NaOH on ice. To encapsulate spheroids in a collagen gel, 25 µl of gel was spread in a flat-bottom 24-well plate (Falcon, 3047) and polymerized for 40 min in a 37°C incubator to form a gel bed. Spheroids were collected with a 1000 µl pipet tip and gently resuspended and mixed with 120-130 µl of ice-cold collagen gel, then deposited onto the gel bed. Spheroid/gel mixture were allowed to polymerized for 1 h in a 37°C, 5% CO_2 incubator, then covered with 1 ml of pre-warmed media and imaged on an inverted Nikon Eclipse T*i* microscope (Nikon, Tokyo, Japan) with automated controls (NIS-Elements; Nikon) and a ×10/0.45 numerical aperture Ph1 objective.

Quantification of cell dissociation from spheroids- Spheroids were visualized in ImageJ and the total number of dissociating cells was recorded up to the specified time.

2D and 3D Alginate Gel Cell Seeding and Imaging- For 2D gel experiments, RGD-coupled alginate with low MW was reconstituted in DMEM (1% penicillin/streptomycin, no FBS) to 2.5% (w/v) and mixed with $CaSO_4$ to form 17 kPa alginate hydrogels. The final Ca^{2+} concentration in the hydrogels was 68.3 mM. The final alginate hydrogels contained 2% (w/v) alginate. After gelling for 45 min, gels were punched to 15 mm disks and the thickness was controlled to 0.4 mm. Gels were transferred to a PDMS mold and customized holders were used to hold the gels at the bottom of the mold. 1 mL of DMEM (1% penicillin/streptomycin, 10% FBS) followed by 100 μL of cell suspension at a concentration of 2×10^4 cells/mL were added to wells containing gels.

For 3D gel experiments, RGD-coupled alginate with low MW was reconstituted in DMEM (1% penicillin/streptomycin, no FBS) to 3% (w/v). First, alginate was mixed with cell suspension, then $CaSO_4$ was added to form 17 kPa alginate hydrogel. The final Ca^{2+} concentration in hydrogels was 68.3 mM. The final alginate hydrogels contained 2% (w/v) alginate. Cell density in the alginate hydrogels was 10^6 cells/mL.

For all gel conditions, cells were cultured at 37°C, 5% CO_2 and incubated overnight before imaging. Gels were imaged on a Nikon A1 confocal microscope (Nikon, Tokyo, Japan) using a 40X water objective with a 1.15 numerical aperture and a resolution of 1024X1024 pixels. 567-nm and 488-nm lasers were used for imaging.

References

1. Chen A, Akhshi TK, Lavoie BD, Wilde A. Importin β2 Mediates the Spatio-temporal Regulation of Anillin through a Noncanonical Nuclear Localization Signal. *J Biol Chem* 2015, **290**(21)**:** 13500-13509.

2. Ratheesh A, Gomez GA, Priya R, Verma S, Kovacs EM, Jiang K*, et al.* Centralspindlin and α-catenin regulate Rho signalling at the epithelial zonula adherens. *Nature Cell Biology* 2012, **14**(8)**:** 818-828.

3. Piekny AJ, Glotzer M. Anillin is a scaffold protein that links RhoA, actin, and myosin during cytokinesis. *Curr Biol* 2008, **18**(1)**:** 30-36.

4. Huff LP, DeCristo MJ, Trembath D, Kuan PF, Yim M, Liu J*, et al.* The Role of Ect2 Nuclear RhoGEF Activity in Ovarian Cancer Cell Transformation. *Genes Cancer* 2013, **4**(11-12)**:** 460-475.

5. Mistriotis P, Wisniewski EO, Bera K, Keys J, Li Y, Tuntithavornwat S*, et al.* Confinement hinders motility by inducing RhoA-mediated nuclear influx, volume expansion, and blebbing. *The Journal of Cell Biology* 2019, **218**(12)**:** 4093-4111.

6. Wisniewski EO, Mistriotis P, Bera K, Law RA, Zhang J, Nikolic M, *et al.* Dorsoventral polarity directs cell responses to migration track geometries. *Science Advances* 2020, **6**(31)**:** eaba6505.

7. Apraiz A, Mitxelena J, Zubiaga A. Studying Cell Cycle-regulated Gene Expression by Two Complementary Cell Synchronization Protocols. *J Vis Exp* 2017(124).

8. Yankaskas CL, Thompson KN, Paul CD, Vitolo MI, Mistriotis P, Mahendra A, *et al.* A microfluidic assay for the quantification of the metastatic propensity of breast cancer specimens. *Nature Biomedical Engineering* 2019, **3:** 452-465.

9. Conrad T, Orom UA. Cellular Fractionation and Isolation of Chromatin-Associated RNA. *Methods Mol Biol* 2017, **1468:** 1-9.

Supplementary Fig. 1 | Uncropped images of Western blots presented in Figure 2e. The dashed red rectangles indicate the area that were cropped and displayed in the figures.

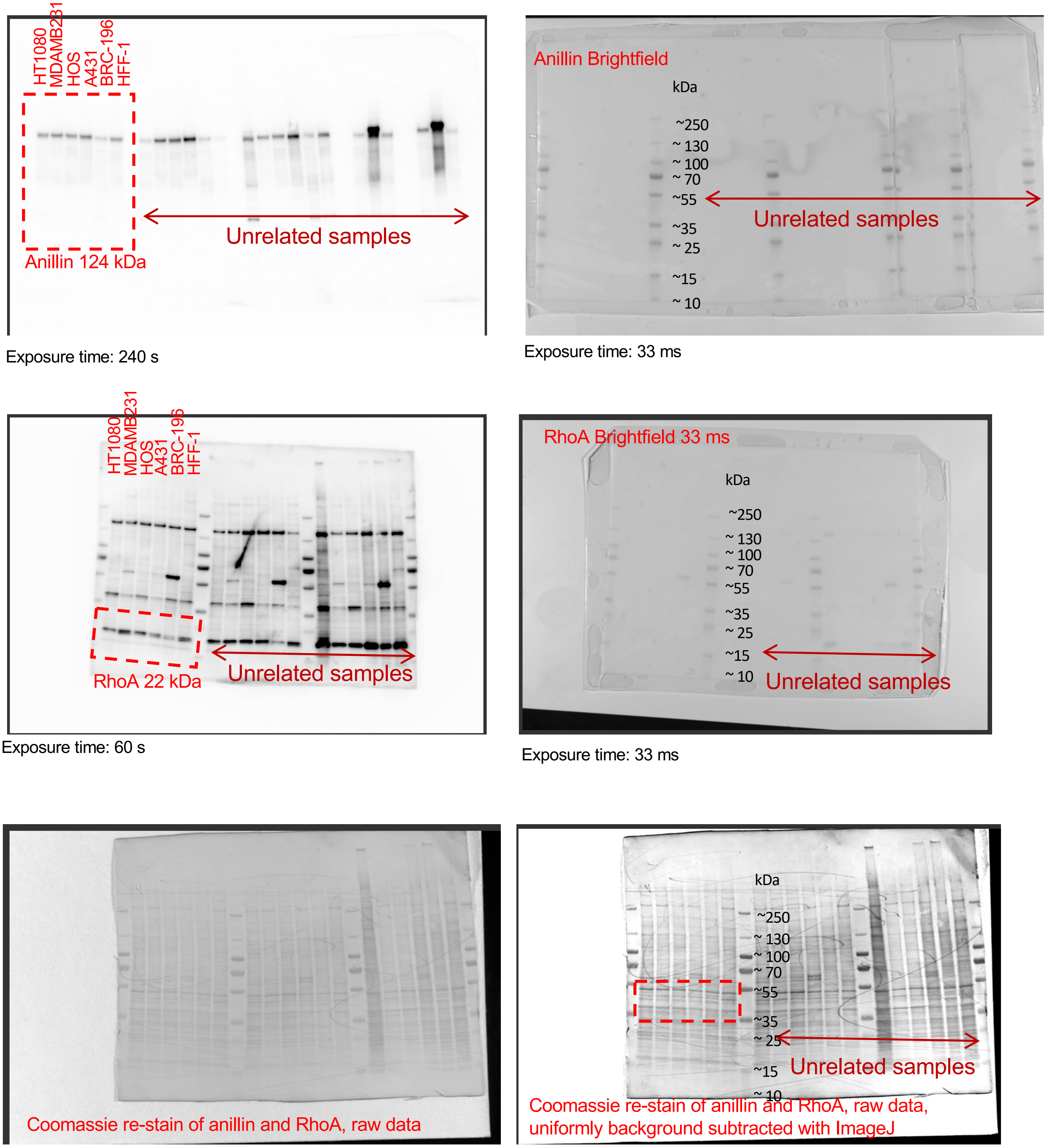

Supplementary Fig. 2 | Uncropped images of Western blots presented in Extended Data Figure 2p. The dashed red rectangles indicate the area that were cropped and displayed in the figures.

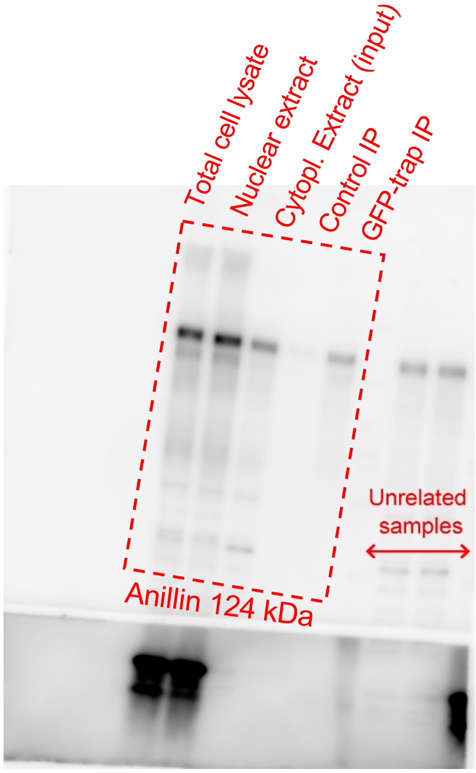

Exposure time: 1260 sec

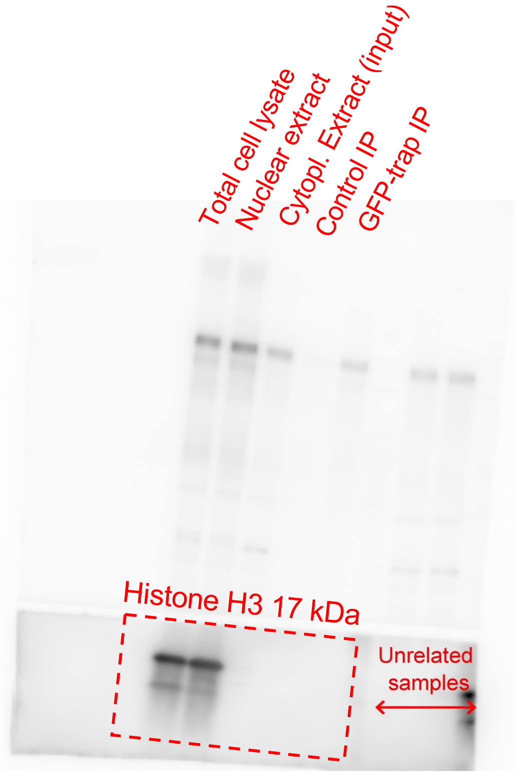

Exposure time: 240 sec

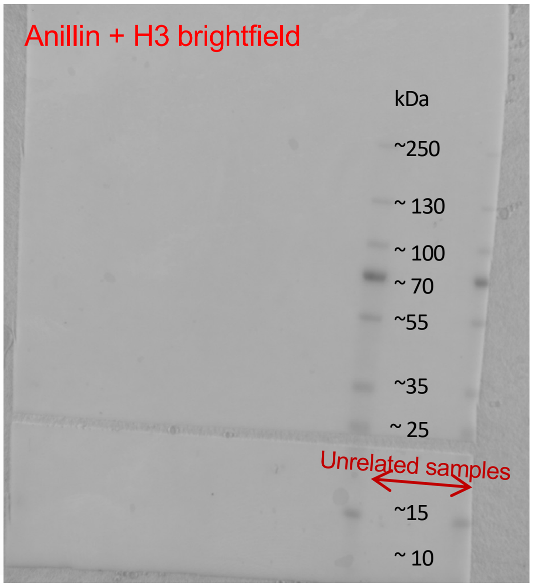

Exposure time: 33 ms

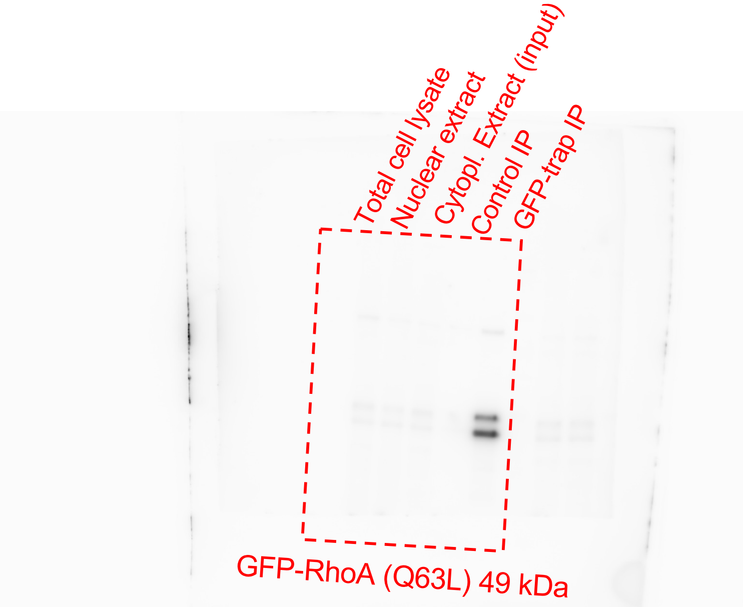

Exposure time: 60 sec

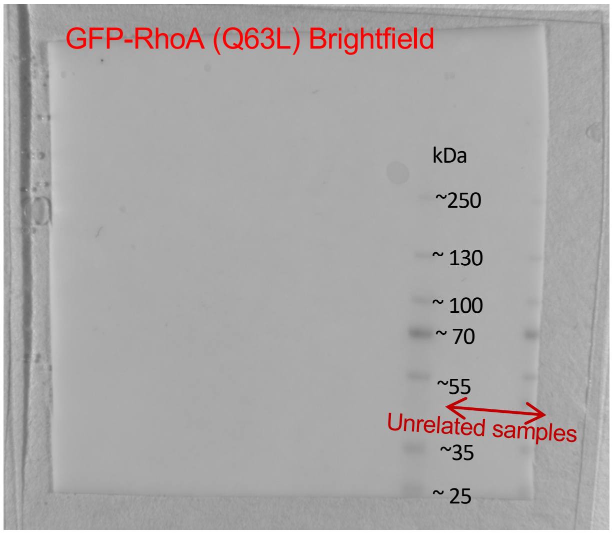

Exposure time: 33 ms

Supplementary Fig. 3 | Uncropped images of Western blots presented in Extended Data Figure 4h. The dashed red rectangles indicate the area that were cropped and displayed in the figures.

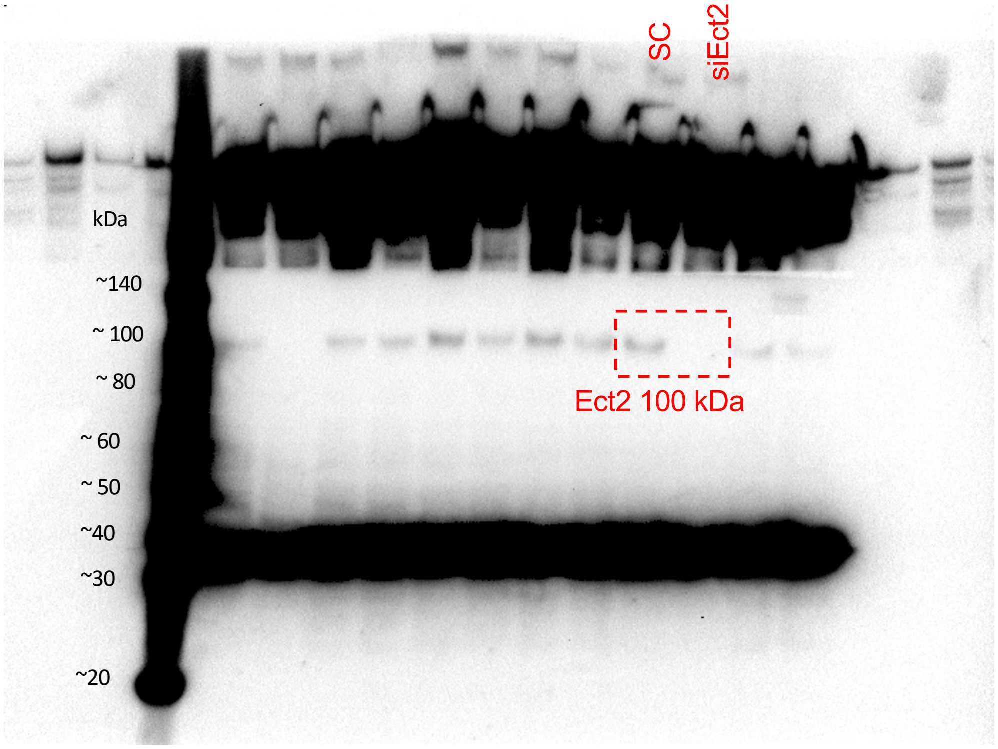

Exposure time: 300 sec

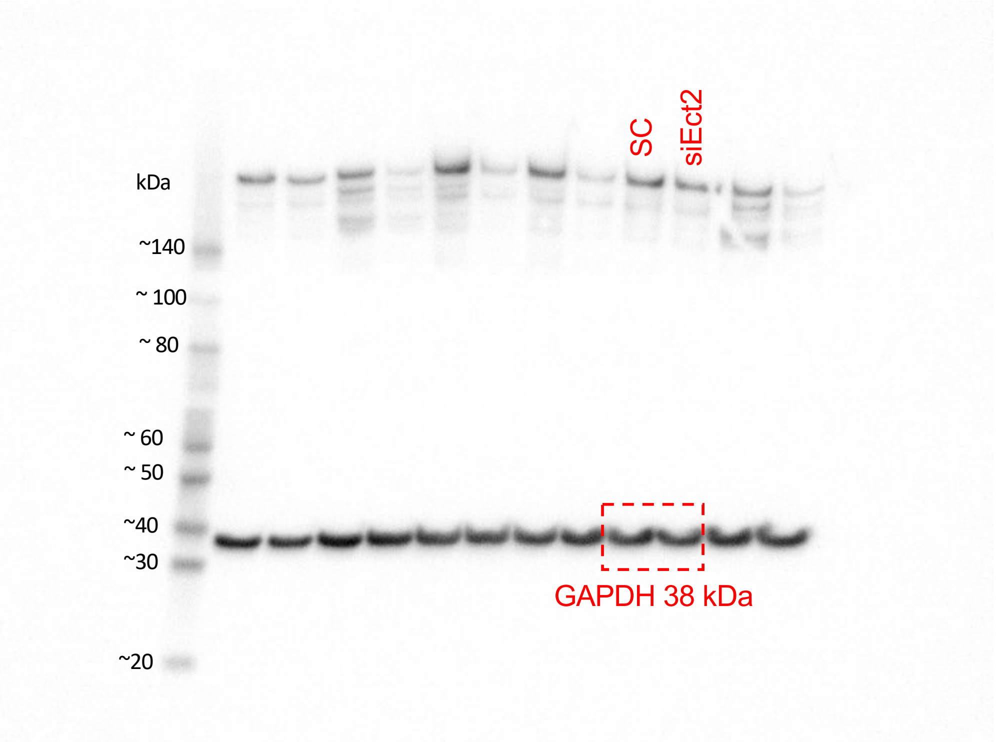

Exposure time: 1 sec

SUPPLEMENTARY VIDEOS

Supplementary Videos 1-4. Colocalization of GFP-anillin and actin at the cell poles during entry into confinement. Representative time-lapse recordings of four GFP-anillin-expressing HT-1080 cells labelled with SPY650-FastAct showing the accumulation and colocalization of anillin and actin, first at the cell rear, then at both poles during cell entry and migration in confining channels. Exposure was uniformly increased to highlight ACEs before cell entry. Scale bars: 10 μm.

Supplementary Video 5. Cells displaying ACEs before their entry in confinement and enrichment after NE rupture. Representative time-lapse recording of an extended G1/S-phase HT-1080 cell expressing GFP-anillin (WT) and NLS-mCherry entering and migrating inside a confining channel. Note the ACEs before cell entry and their enrichment following NE rupture. Exposure was uniformly increased to highlight ACEs before cell entry. Scale bars: 10 μm.

Supplementary Video 6. Cells in confinement experiencing multiple NE ruptures. Representative time-lapse recording of an extended G1/S-phase HT-1080 cell expressing GFP-anillin (WT) and NLS-mCherry inside a confining channel. Exposure was uniformly increased to highlight ACEs. Scale bar: 10 μm.

Supplementary Video 7. Initiation of NLS-mCherry leakage correlated with further anillin enrichment to the cell cytoplasm and membrane *in vivo*. Representative time-lapse recording of a confined HT-1080 cell localized near the tumor edge displaying front and rear ACEs that are further enriched upon NE rupture, as shown by GFP-anillin (top, purple heatmap LUT) and NLS-mCherry (bottom, RGB rainbow LUT) exit from the nucleus and localization to the cytosol. Purple arrowheads, anillin on the plasma membrane and transient decrease of NLS intensity in the nucleus and increase in the cytoplasm. Scale bar: 20 μm.

Supplementary Videos 8-10. Anillin accumulation on the plasma membrane at cell front and/or rear without signs of clear NE rupture *in vivo*. Representative time-lapse recordings of confined HT-1080 cells localized near the tumor edge displaying front and rear ACEs even without NE ruptures, as shown by GFP-anillin (top, purple heatmap LUT) and NLS-mCherry (bottom, RGB rainbow LUT). Purple arrowheads show anillin on the plasma membrane. Scale bar: 20 μm.

Supplementary Video 11. Nuclear GFP-Ect2 exits to the cytoplasm in confinement following NE ruptures. Representative time-lapse recording of an HT-1080 cell expressing GFP-Ect2 and NLS-mCherry entering and migrating inside confining channels, showing accumulation of GFP-Ect2 in the cytoplasm as NE ruptures. Scale bars: 10 μm.

Supplementary Video 12. The impact of anillin on cell invasion *in vivo*. mCherry-tagged HT-1080 cells expressing GFP-anillin-Δ3 and HA-Ect2-DHmut dual mutants formed more compact and less invasive lesions than GFP-anillin (WT)/HA-Ect2(WT) controls. Upper panels show mCherry and GFP channels; lower panels show GFP channel only. 20 min/frame; 7.6 h total duration; 10x magnification. Scale bar: 50 μm.
