# Supplementary material for: Cytoplasmic anillin and Ect2 promote RhoA/myosin II-dependent confined migration and invasion
Source: Nat Mater. 2025 Jun 26;24(9):1476–88. doi: 10.1038/s41563-025-02269-9 (PMC12404997; doi:10.1038/s41563-025-02269-9)
Supplement: Supplementary file 2 — Reporting Summary [file 41563_2025_2269_MOESM2_ESM.pdf]

Corresponding author(s): Konstantinos Konstantopoulos

Last updated by author(s): Apr 9, 2025

Reporting Summary

Nature Portfolio wishes to improve the reproducibility of the work that we publish. This form provides structure for consistency and transparency in reporting. For further information on Nature Portfolio policies, see our Editorial Policies and the Editorial Policy Checklist.

Statistics

For all statistical analyses, confirm that the following items are present in the figure legend, table legend, main text, or Methods section.

n/a	Confirmed	
☐	☒	The exact sample size (*n*) for each experimental group/condition, given as a discrete number and unit of measurement
☐	☒	A statement on whether measurements were taken from distinct samples or whether the same sample was measured repeatedly
☐	☒	The statistical test(s) used AND whether they are one- or two-sided<br>*Only common tests should be described solely by name; describe more complex techniques in the Methods section.*
☐	☒	A description of all covariates tested
☐	☒	A description of any assumptions or corrections, such as tests of normality and adjustment for multiple comparisons
☐	☒	A full description of the statistical parameters including central tendency (e.g. means) or other basic estimates (e.g. regression coefficient) AND variation (e.g. standard deviation) or associated estimates of uncertainty (e.g. confidence intervals)
☐	☒	For null hypothesis testing, the test statistic (e.g. *F*, *t*, *r*) with confidence intervals, effect sizes, degrees of freedom and *P* value noted<br>*Give P values as exact values whenever suitable.*
☒	☐	For Bayesian analysis, information on the choice of priors and Markov chain Monte Carlo settings
☒	☐	For hierarchical and complex designs, identification of the appropriate level for tests and full reporting of outcomes
☐	☒	Estimates of effect sizes (e.g. Cohen's *d*, Pearson's *r*), indicating how they were calculated

Our web collection on statistics for biologists contains articles on many of the points above.

Software and code

Policy information about availability of computer code

Data collection	Data were collected using standard features of NIS-Elements (Version 5.42.01 or Version 3.22.00), Image Lab (Version 6.1.0 build 7), MetaXpress (Version 6.7.1.157), SymPhoTime 64 (PicoQuant), ZenBlue (Zen 3.11).
Data analysis	Data were organized primarily using Microsoft Excel (Version 2310 Build 16.0.16924.20054) and Graphpad Prism (Version 6,7,8,9,10). Select analysis were performed in ImageJ (Version 1.54f), MATLAB (Version R2023b), and MetaXpress (Version 6.7.1.157) as described in Methods. Custom codes used for nuclear morphology analysis and FLIM pseudocolor display are available at DOI: 10.5281/zenodo.15178932.

For manuscripts utilizing custom algorithms or software that are central to the research but not yet described in published literature, software must be made available to editors and reviewers. We strongly encourage code deposition in a community repository (e.g. GitHub). See the Nature Portfolio guidelines for submitting code & software for further information.

Data

Policy information about availability of data

All manuscripts must include a data availability statement. This statement should provide the following information, where applicable:

- Accession codes, unique identifiers, or web links for publicly available datasets
- A description of any restrictions on data availability
- For clinical datasets or third party data, please ensure that the statement adheres to our policy

The main data supporting the results of this study are available within the paper, its Extended Data and Supplementary Figure files. Also, source data files are provided. The custom codes used for nuclear morphology analysis and FLIM pseudocolor display are available at https://github.com/pixel-bio/Cell-in-Channel-anal

Research involving human participants, their data, or biological material

Policy information about studies with human participants or human data. See also policy information about sex, gender (identity/presentation), and sexual orientation and race, ethnicity and racism.

Reporting on sex and gender	N/A
Reporting on race, ethnicity, or other socially relevant groupings	N/A
Population characteristics	N/A
Recruitment	N/A
Ethics oversight	N/A

Note that full information on the approval of the study protocol must also be provided in the manuscript.

Field-specific reporting

Please select the one below that is the best fit for your research. If you are not sure, read the appropriate sections before making your selection.

☒ Life sciences ☐ Behavioural & social sciences ☐ Ecological, evolutionary & environmental sciences

For a reference copy of the document with all sections, see nature.com/documents/nr-reporting-summary-flat.pdf

Life sciences study design

All studies must disclose on these points even when the disclosure is negative.

Sample size	We indicated in figure legends the sample size, number of replicates and p value for each experiment. No predetermination of sample size was done. Sample size was chosen based on the throughput of the technique used. Sample sizes were sufficient to show the same trends between the replicates performed for each experiment, and by statistical testing. For animal experiments, no statistical methods were used to calculate sample size and group size, and the sample size was determined based on experience of similar assays performed earlier. In each experiment multiple cells or animals were examined in parallel leading to sample sizes primarily of the order of tens to hundreds.
Data exclusions	Data were excluded for in vitro cell entry experiments based on pre-established criteria: dividing cells or cells that entered immediately following mitosis were excluded from analysis. For mouse IVM, no mice were excluded. For quantification of anillin in chick embryo experiments, cells that do not have visible amount of both GFP and mCherry fluorescence signal, or whose nuclei were indistinguishable, or are clearly undergoing cell division, are excluded from the analysis; one tumor was excluded for quantification of percentage of cells with ACEs due to poor image quality.
Replication	The number of replicates for each experiment has been indicated in the figure legends. Most experiments were repeated 3 or more times, with similar results observed each time. Select control experiments were repeated 2 times with consistent data across all replicates.
Randomization	For in vitro fluorescence quantification, cells positive for their respective fluorescence markers were randomly selected for analysis. Mice were randomized based on weight to maintain similar average weight across experimental groups. For CAM extravasation assays, embryos were randomly divided into experimental groups prior to injection. For all other experiments, cells were randomly distributed into experimental groups before imaging and analysis.
Blinding	Mice injections and chick embryo experiments were performed in a blinded manner, without prior knowledge of cell line identity or expected results. For the remaining experiments, researchers were not blinded as data collection and analysis were performed by the same individual assigning the groups. Wherever possible findings (e.g., cell migration speeds, cell number, fluorescence intensity) were analyzed in an unbiased manner either manually or by the use of automated Fiji and custom analysis codes in Matlab.

Reporting for specific materials, systems and methods

We require information from authors about some types of materials, experimental systems and methods used in many studies. Here, indicate whether each material, system or method listed is relevant to your study. If you are not sure if a list item applies to your research, read the appropriate section before selecting a response.

Materials & experimental systems

n/a	Involved in the study
☐	☒ Antibodies
☐	☒ Eukaryotic cell lines
☒	☐ Palaeontology and archaeology
☐	☒ Animals and other organisms
☒	☐ Clinical data
☒	☐ Dual use research of concern
☒	☐ Plants

Methods

n/a	Involved in the study
☒	☐ ChIP-seq
☐	☒ Flow cytometry
☒	☐ MRI-based neuroimaging

Antibodies

Antibodies used	Primary antibodies used for immunofluorescence were: rabbit anti-anillin (Sigma-Aldrich, HPA005680; lot A104338; 1:1000), mouse anti-RhoA (Santa Cruz Biotechnology, sc-418; lot H1122; 1:200), rabbit anti-Ect2 (EMD Millipore, 07-1364; lot 3979351; 1:100), mouse anti-HA (Cell Signaling, 2367; lot 5; 1:600), rabbit anti-HA (Cell Signaling, 3724; lot 11; 1:100), rabbit anti-phospho-myosin light chain 2 (Cell Signaling, 3671; lot 6; 1:50). Secondary antibodies used for IF were: Alexa Fluor 488 donkey anti-rabbit (Invitrogen, A32790n, 1:1000), Alexa Fluor 555 Donkey anti-mouse (Invitrogen A32773, 1:1000), Alexa Fluor 488 goat anti-rabbit immunoglobulin G (IgG) H+L, (Invitrogen; A11034; Lot 2256692; 1:200), Alexa Fluor 488 goat anti-mouse IgG (H+L) (Invitrogen, A11001; Lot 2486523; 1:200), Alexa Fluor Plus 647 goat anti-rabbit immunoglobulin G (IgG) H+L, (Invitrogen; A21245; Lot 2299231; 1:200), Alexa Fluor 568 goat anti-rabbit (H+L) (Invitrogen, A11011; Lot 1558746; 1:200).<br>Primary antibodies used for western blotting were: rabbit anti-anillin (Sigma-Aldrich, HPA005680; lot A104338; 1:1000), mouse anti-RhoA (Santa Cruz Biotechnology, sc-418; lot H1122; 1:200), rabbit anti-Histone H3 (Cell Signaling, 4499; lot 9; 1:2000), mouse anti-Ect2 (Santa Cruz, sc-514750; lot K0718; 1:100), mouse anti-GFP (Roche, 11814460001; lot 54732800; 1:1000). Rabbit anti-GAPDH was used as a loading control (Cell Signaling, 2118S; Lot 14; 1:1000). Secondary antibodies used for western blotting were: goat anti-mouse IgG, HRP-linked antibody (Cell Signaling, 7076; lot 36,38; 1:2000) and goat anti-rabbit IgG, HRP-linked antibody (Cell Signaling, 7074; lot 31,32; 1:2000).
Validation	Prior to purchasing, antibody validations were performed by the manufacturer and available on their website:<br>www.sigmaaldrich.com/US/en/coa/SIGMA/HPA005680/A104338<br>datasheets.scbt.com/sc-418.pdf<br>https://www.emdmillipore.com/US/en/product/Anti-ECT2-Antibody,MM_NF-07-1364#anchor_COA<br>https://www.cellsignal.com/products/14031/datasheet?images=1&protocol=0<br>www.cellsignal.com/datasheet.jsp?productId=3671&images=1<br>www.cellsignal.com/datasheet.jsp?productId=4499&images=1<br>datasheets.scbt.com/sc-514750.pdf<br>https://www.sigmaaldrich.com/US/en/coa/ROCHE/11814460001/54732800<br>www.cellsignal.com/datasheet.jsp?productId=2118&images=1<br>www.thermofisher.com/order/genome-database/dataSheetPdf?<br>producttype=antibody&productsubtype=antibody_secondary&productId=A-32790&version=359<br>www.thermofisher.com/order/genome-database/dataSheetPdf?<br>producttype=antibody&productsubtype=antibody_secondary&productId=A32773&version=359<br>www.thermofisher.com/order/genome-database/dataSheetPdf?<br>producttype=antibody&productsubtype=antibody_secondary&productId=A-11034&version=359<br>www.thermofisher.com/order/genome-database/dataSheetPdf?<br>producttype=antibody&productsubtype=antibody_secondary&productId=A-11001&version=359<br>www.thermofisher.com/order/genome-database/dataSheetPdf?<br>producttype=antibody&productsubtype=antibody_secondary&productId=A21245&version=359<br>www.thermofisher.com/order/genome-database/dataSheetPdf?<br>producttype=antibody&productsubtype=antibody_secondary&productId=A-11011&version=359<br>www.cellsignal.com/datasheet.jsp?productId=7076&images=1<br>www.cellsignal.com/datasheet.jsp?productId=7074&images=1<br>Additionally, antibodies for western blotting were verified based on the appropriate molecular weight of the protein probed and for immunofluorescence by comparison of their cellular distribution to that provided in the manufacturer's datasheet.

Eukaryotic cell lines

Policy information about cell lines and Sex and Gender in Research

Cell line source(s)	MDA-MB-231, A431, HFF-1, and HT1080 were purchased from American Type Culture Collection (ATCC). Human osteosarcoma (HOS) cells were obtained from the NIH AIDS Research and Reference Reagent Program (Division of AIDS, NIAID, NIH, Betheshda, MD). BRC-196 cells were provided by Dr. Peter Siegel (McGill University). Select cell lines were

	modified from the parental line, as described in Methods (e.g., development of cell lines with scramble, shRNA or live reporter)
Authentication	Cell lines were initially authenticated by ATCC. Cell morphology was routinely monitored to ensure proper cell state and confirm authentication.
Mycoplasma contamination	All cell lines were regularly tested by PCR and verified to be free of mycoplasma contamination.
Commonly misidentified lines (See ICLAC register)	No commonly misidentified cell lines were used.

Animals and other research organisms

Policy information about studies involving animals; ARRIVE guidelines recommended for reporting animal research, and Sex and Gender in Research

Laboratory animals	For mouse IVM, athymic nu/nu female mice were obtained from the Department of Experimental Radiation Oncology, M.D. Anderson Cancer Center. Dorsal skin-fold chambers were mounted on 8 to 12-week-old female athymic nu/nu mice. Animal cages were maintained at 40%-55% relative humidity, a temperature of 21°C with a 12-hour light cycle, and a minimum of 10 room air changes per hour. For mice carrying the skin-fold chamber, cages were maintained at 28°C. Cages were changed once a week. Mice were fed a diet containing low fiber (5%), protein (20%) and fat (5-10%).<br><br>For chick embryo, fertilized White Leghorn chicken eggs were acquired from the University of Alberta Poultry Research Centre and maintained at 38°C. Embryos were isolated from their shells after 4 days of incubation and maintained under shell-less conditions in a covered dish placed in an air incubator at 38°C and 60% humidity.
Wild animals	This study did not involve wild animals.
Reporting on sex	Female mice were used in this study.
Field-collected samples	This study did not involve samples collected from the field.
Ethics oversight	Mouse IVM studies were performed by following procedures approved by Association for Assessment and Accreditation for Laboratory Animal Care (AAALAC) at M.D. Anderson. Chick embryo studies were performed by following procedures approved by the University of Alberta Institutional Animal Care and Use Committee.

Note that full information on the approval of the study protocol must also be provided in the manuscript.

Plants

Seed stocks	*Report on the source of all seed stocks or other plant material used. If applicable, state the seed stock centre and catalogue number. If plant specimens were collected from the field, describe the collection location, date and sampling procedures.*
Novel plant genotypes	*Describe the methods by which all novel plant genotypes were produced. This includes those generated by transgenic approaches, gene editing, chemical/radiation-based mutagenesis and hybridization. For transgenic lines, describe the transformation method, the number of independent lines analyzed and the generation upon which experiments were performed. For gene-edited lines, describe the editor used, the endogenous sequence targeted for editing, the targeting guide RNA sequence (if applicable) and how the editor was applied.*
Authentication	*Describe any authentication procedures for each seed stock used or novel genotype generated. Describe any experiments used to assess the effect of a mutation and, where applicable, how potential secondary effects (e.g. second site T-DNA insertions, mosiacism, off-target gene editing) were examined.*

Flow Cytometry

Plots

Confirm that:

☒ The axis labels state the marker and fluorochrome used (e.g. CD4-FITC).

☒ The axis scales are clearly visible. Include numbers along axes only for bottom left plot of group (a 'group' is an analysis of identical markers).

☒ All plots are contour plots with outliers or pseudocolor plots.

☒ A numerical value for number of cells or percentage (with statistics) is provided.

Methodology

Sample preparation	800,000 cells of HT-1080 GFP-anillin were synchronized as described in Methods, kept in DMEM 2.5% FBS (early G1/S) or DMEM 2.5% 8 mM hydroxyurea (extended G1/S) for 5 h, trypsinized, centrifuged, and resuspended in 500 µl D-PBS. Samples were briefly kept on ice, then fixed with 4.5 ml of ice-cold 70% ethanol. To stain with propidium iodide (PI), samples were centrifuged at 300 g, 4oC for 5 min, washed with 5 ml ice-cold D-PBS, centrifuged again and incubated for 30 min at 37oC in

	2x106 cells/ml of staining solution: 20 µg/ml PI (Sigma P4864), 200 µg/ml RNase A (Invitrogen, 8003089), 0.1% Triton X-100 (Sigma, T9284) in PBS.
Instrument	BD FACSCanto
Software	FlowJo v10
Cell population abundance	After gating, >98% cell population were PI-positive.
Gating strategy	A polygonal FSC-A (65,000-250,000) vs SSC-A (5,000-100,000) gate was used to filter out debris. Single cells were gated with a polygonal FSC-A (50,000-220,000) vs FSC-H (40,000-220,000) gate. A square PI-A (52,000 - 160,000) vs PI-W (64,000-158,000) was used to isolate PI-positive subpopulations.

☒ Tick this box to confirm that a figure exemplifying the gating strategy is provided in the Supplementary Information.
